# Eco-technological potential of salinity-driven functional specialization in Indian solar salterns revealed by integrated culturomics and whole-metagenome profiling

**DOI:** 10.1186/s12866-026-05253-8

**Published:** 2026-06-20

**Authors:** Shubham Pandey, Bhavna Parmar, Anjali Gupta, Ayushi Singh, Ashwini Chauhan, Kuo-Wei Huang, Ram Karan

**Affiliations:** 1 Department of Microbiology, University of Delhi South Campus, 110021 New Delhi, India; 2 Center for Renewable Energy and Storage Technologies (CREST), Physical Science and Engineering Division, KAUST Catalysis Center (KCC), King Abdullah University of Science and Technology, Thuwal, 23955 Saudi Arabia

**Keywords:** Hypersaline microbiomes, Osmoadaptation, Whole-metagenome sequencing, Biosynthetic gene clusters, Extremophiles, Extremozymes, Microbial dark matter

## Abstract

Solar salterns are environmentally stable yet biologically extreme ecosystems that serve as vital models for understanding and managing hypersaline environments, including industrial saline effluents. Despite their ecological and biotechnological significance, Indian solar salterns remain functionally underexplored. In this study, we integrated culture-dependent isolation with whole-metagenome sequencing to investigate microbial community assembly, functional specialization, and eco-technological potential across four geographically distinct Indian salterns.

Physicochemical analyses revealed pronounced spatial variation in salinity, pH, and electrical conductivity, which together strongly structured microbial communities. Metagenomic sequencing generated between 4.84 and 8.68 Gb of raw data across individual site, yielding between 429,420 and 669,991 predicted genes in high-salinity locations. Taxonomic reconstruction demonstrated archaeal dominance at extreme salinity, particularly among Euryarchaeota, whereas comparatively moderate salinity sites supported more balanced bacterial–archaeal assemblages. Alpha diversity patterns indicated higher richness in Tamil Nadu and Rajasthan, while Gujarat exhibited reduced evenness consistent with environmental filtering.

Culture-dependent approaches recovered 42 halophilic and polyextremophilic isolates, primarily affiliated with Halobacteriaceae and Bacillaceae, complementing the broad taxonomic detection of these lineages inferred from metagenomic data. Functional annotation revealed extensive enrichment of genes involved in ion transport, energy production, osmoprotectant biosynthesis, and DNA repair, reflecting an adaptive mechanism critical for survival in high-salinity industrial processes. Amino acid metabolism genes exceeded 25,000 hits in selected sites, and replication and repair genes reached 32,554 in Gujarat, indicating heightened stress-response activity. Secondary metabolite biosynthetic gene clusters, including pathways for novel antimicrobial peptides, terpene, ribosomally synthesized and post-translationally modified peptide-like, and type III polyketide synthase pathways, were widely distributed, offering new biological control mechanisms for environments impaired by stress. Antimicrobial resistance signatures were limited and unevenly distributed across sites.

These findings demonstrate that salinity acts as a dominant ecological filter driving both taxonomic composition and functional specialization in Indian solar salterns. By linking environmental gradients to adaptive genomic traits, this study establishes a functional baseline for hypersaline ecosystems.

**Supplementary Information:**

The online version contains supplementary material available at https://doi.org/10.1186/s12866-026-05253-8.

## Introduction

Extremophilic microorganisms constitute a distinctive and functionally important component of the biosphere, thriving in environments once considered incompatible with life. Their ability to maintain structural integrity and catalytic efficiency under extremes of salinity, temperature, pressure, and pH has attracted sustained interest, particularly for applications requiring robust enzymes and metabolites capable of operating under harsh industrial conditions [1–5]. Concrete global examples of this eco-technological potential include integrating halophilic lipases into high-efficiency laundry detergents, using salt-tolerant proteases to accelerate high-salinity food fermentation, and using extreme-tolerant biocatalysts for the remediation of hypersaline industrial effluents [6–8]. As a result, extremophiles have become central to diverse biotechnological sectors, supporting industrial catalysis, waste remediation, and biomedical innovations, including drug delivery systems and stress-tolerant bioprocesses [9–16].

Despite their recognized importance, experimental access to extremophilic microorganisms remains limited. Traditional culture-dependent approaches have played a crucial role in establishing microbial physiology and taxonomy, yet they capture only a small fraction of existing diversity. It is widely estimated that less than 0.001–0.1% of prokaryotic species have been successfully cultivated under laboratory conditions, leaving the vast majority of microbial lineages unexplored (microbial dark matter) [17–19]. Many extremophiles depend on complex physicochemical gradients, community interactions, or long-term environmental stability that are difficult to reproduce *ex situ*. These constraints not only limit taxonomic characterization but also hinder the identification and prospective utilization of novel genes, enzymes, and metabolic pathways with potential industrial relevance [18, 20].

Metagenomics has emerged as a powerful strategy to overcome these limitations by enabling direct access to the collective genetic material of microbial communities. By bypassing cultivation requirements, metagenomic approaches provide simultaneous insight into community composition and functional capacity, facilitating the discovery of genes involved in stress adaptation, metabolic versatility, and secondary metabolite biosynthesis [21–25]. Over the past decade, metagenomic investigations have substantially expanded our understanding of microbial life in extreme environments such as hydrothermal vents, polar lakes, hypersaline flats, and deep brine systems, revealing both ecological structuring and evolutionary strategies employed by extremophilic taxa [22, 26–29].

The rapid development of next-generation sequencing (NGS) technologies has further transformed extremophile research by enabling high-throughput sequencing of complex environmental DNA at unprecedented depth. These technologies allow the detection and analysis of rare and low-abundance community members that are often overlooked by conventional approaches [26, 30, 31]. High-resolution metagenomic datasets now support genome reconstruction, comparative functional annotation, and metabolic inference, thereby accelerating the identification of enzymes and metabolites suitable for industrial and environmental applications [32, 33]. In parallel, the expansion of curated metagenomic repositories has enabled cross-ecosystem comparisons, linking genetic traits to geochemical parameters and revealing convergent strategies of microbial adaptation across extreme habitats [27, 34, 35].

Within this broader context, hypersaline solar salterns represent particularly compelling systems for studying extremophilic microbial communities. These environments are characterized by sustained high salinity, intense solar radiation, and pronounced ionic stress, leading to the dominance of highly specialized halophilic microorganisms, often enriched in archaeal lineages [36, 37]. While salterns in regions all over the globe have been investigated in considerable detail, including foundational metagenomic studies from the Santa Pola salterns in Spain, Ethiopian soda lakes such as Lake Chitu and Shala, and the Great Salt Lake, Indian solar salterns remain comparatively underrepresented in functional metagenomic studies [38–44]. Furthermore, due to the intense osmotic stress and competitive pressures that drive horizontal gene transfer, these hypersaline environments are increasingly recognized as critical yet understudied environments potentially harboring antimicrobial resistance (AMR) and virulence genes [45, 46]. Given India’s extensive coastline and diverse climatic regimes, its salterns offer a unique opportunity to examine how regional physicochemical variability shapes microbial community structure and functional potential.

**Fig. 1:**
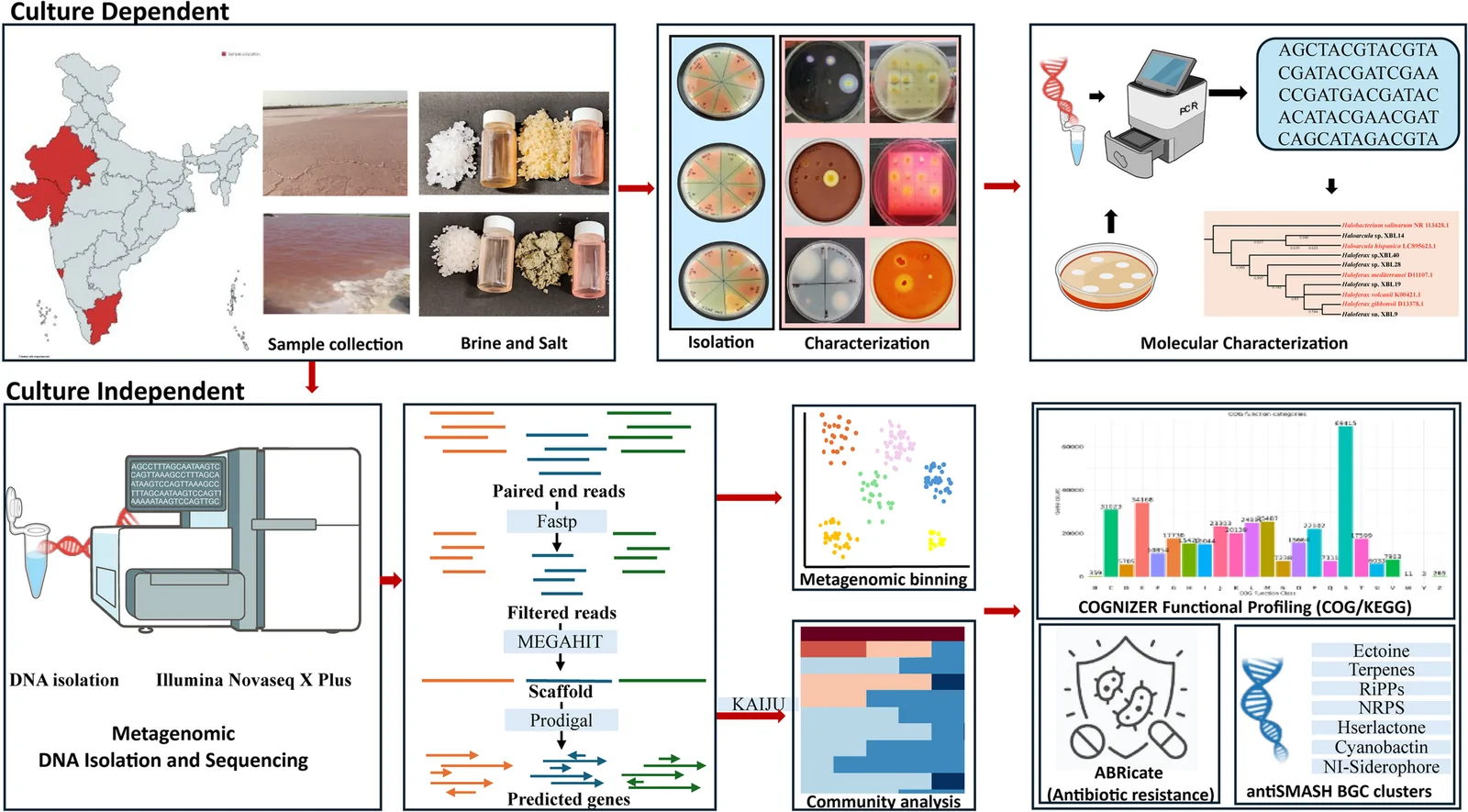
Schematic representation of the polyphasic workflow used in this study. The upper panel depicts the culture-dependent isolation and screening strategy. The lower panel illustrates the culture-independent metagenomic pipeline

In the present study, we investigated microbial communities from four geographically and environmentally distinct Indian solar salterns: Naravakkam (Tamil Nadu), Sambhar Salt Lake (Rajasthan), Betim Salt Pan (Goa), and Bhimrana, Mithapur (Gujarat). By integrating physicochemical characterization with culture-dependent isolation and whole-metagenome sequencing, we aimed to (i) assess site-specific microbial diversity and community organization, (ii) characterize functional gene repertoires associated with hypersaline adaptation, (iii) identify enzymes and biosynthetic pathways of potential industrial relevance (e.g., halophilic lipases for laundry detergents and salt-tolerant proteases for food fermentation [6–8]), and (iv) evaluate the distribution of antimicrobial resistance and virulence-associated genes in these environments (Fig. 1). This combined approach provides a comprehensive functional genomic framework for Indian saltern microbiomes and highlights these extreme habitats as underexplored repositories of extremozymes, bioactive compounds, and resistance determinants.

## Materials and methods

### Site description, sampling strategy, and physicochemical characterization

To capture robust extremophilic communities from historically established, highly saline regions across India, salt, soil, and water samples were collected from Naravakkam (Tamil Nadu, 12.20°N, 79.96°E), Sambhar Salt Lake (Rajasthan, 26.92°N, 75.04°E), Betim Salt Pan, Tiswadi Taluka (Goa, 15.45°N, 73.88°E), and Bhimrana, Mithapur (Gujarat, 22.24°N, 69.00°E), representing coastal evaporation pans (Naravakkam, Betim), an inland hypersaline lake (Sambhar), and an industrial seawater crystallizer (Bhimrana). At each site, three replicate samples per matrix (sediment, salt crust, brine) were collected during November 2023–March 2024 along a ~ 10 m transect and pooled into one composite per matrix. Sediments and salt crusts were sampled from the upper 0–5 cm using sterile stainless-steel spatulas, and brine (1 L) was collected in sterile bottles. Samples were transported on ice, with a working aliquot stored at 4 °C for culture isolation (processed within 72 h) and an aliquot stored at − 80 °C until DNA extraction. In situ physicochemical parameters were recorded at the time of sampling. Temperature was measured using a digital thermometer (DMT-101), and pH was determined using an Eutech pH700 meter (Thermo Fisher Scientific) calibrated with standard buffer solutions. The liquid brine sample was analysed in its native state to preserve true in situ physicochemical parameters. For the three solid salt samples, a 1% (w/v) solution was prepared to ensure a uniform mass-to-volume suspension, providing a consistent baseline density for accurate assessment of the sample properties. All final reported values were subsequently corrected using the appropriate dilution factors to reflect the exact in situ parameters. To accurately capture salinity profiles across varying sample matrices, electrical conductivity (EC) was measured with a Labtronics LT-16 Digital Conductivity Meter using two distinct protocols. For environmental liquid samples (Tamil Nadu brine), EC was measured directly. For solid sediment and crystalline salt samples, EC was determined using a standard 1:100 solid-to-water aqueous extract (EC₁:₁₀₀), prepared by dissolving 1 g of the environmental sample in 100 mL of AMQ water.

### Enrichment and isolation of halophilic and polyextremophilic microbes

Halophilic and polyextremophilic microbes were isolated using established complex moderate halophilic media (MHM) agar and extreme halophilic media (EHM) agar modified with 17% (w/v) and 23% (w/v) NaCl, respectively, for enrichment [38]. These complex media utilize yeast extract (10.0 g/L) and peptone (5.0 g/L) to provide the necessary carbon sources, trace elements, and vitamins for robust extremophile growth, supplemented with KCl (2.0 g/L). To accurately reflect the diverse, localized microenvironments and prevent pH drift or salt precipitation during sterilization, the basal medium was custom adjusted to match each sample’s in situ pH before autoclaving. Concentrated stock solutions of MgSO_4_·7H_2_O (20.0 g/L) and CaCl_2_·2H_2_O (0.36 g/L) were adjusted to the corresponding pH, sterilized independently, and added to the basal medium post-autoclaving.

For plating, a 10^− 1^ dilution of soil, salt, and water samples was prepared using sterile liquid medium (without agar) as the diluent to match osmotic pressure and prevent osmotic shock. The entire dilution was spread-plated in duplicate on the above medium and incubated aerobically at 40 °C for 15–30 days. This specific methodology, combining 40 °C (reflecting the average environmental site temperatures) with prolonged incubation, was deliberately employed as an enrichment strategy to favor the recovery of extreme halophilic archaea. Resultant colonies were purified by repeated streaking on MHM agar and EHM agar. Purified isolates were stored in agar slants at 4 °C and subcultured every 15 days.

### Morphological, biochemical, and molecular characterization of isolates

All isolates were examined for colony characteristics, morphology, Gram reaction, microscopic cell structure, and cell arrangements. Gram staining procedure was performed [47] with a 2% (v/v) acetic acid pre-fixation step for haloarchaeal isolates to preserve cell integrity under low-salt staining conditions [48]. While a preliminary morphological assessment included standard Gram staining, definitive taxonomic assignment relied exclusively on 16S rRNA gene sequencing due to the inherent unreliability of Gram staining for archaea, which lack a standard peptidoglycan cell wall. Isolates were screened (*n* = 3) for amylase, esterase, protease, lipase, xylanase, and cellulase activities by inoculating 3–4 days old cultures (corresponding to their mid-to-late exponential growth phase) onto agar media supplemented with specific substrates: 1% w/v starch (amylase), 1% v/v Tween 80 (esterase), 1% w/v skim milk powder (protease), 1% v/v tributyrin with 0.01% w/v phenol red (lipase), 0.1% w/v birchwood xylan (xylanase), and 1% w/v carboxymethyl cellulose (cellulase). Plates were incubated at 40 °C for 4–7 days in triplicate. Enzymatic activity was identified by the formation of clear halos (esterase, protease), yellow zones (lipase), or clearance zones following staining with Gram’s iodine (amylase) or 0.5% w/v Congo red followed by 5 M NaCl washing (xylanase and cellulase) [38]. Clearance zones were measured manually using a standard millimetre scale. To standardize against variations in microbial growth, actual enzymatic activity zones were calculated by subtracting the colony diameter from the total halo diameter. All assay results are expressed as mean ± standard deviation.

Representative isolates displaying distinct phenotypic traits were subjected to genomic DNA extraction using a modified CTAB method [49]. The purified DNA was then used to amplify and sequence the 16S rRNA gene to confirm their taxonomic identity, incorporating repeated, stringent 70% ethanol washes to effectively remove co-precipitating halophilic salts and exopolysaccharides. DNA purity and concentration were assessed spectrophotometrically (A_260_/A_280_ ratio), and integrity was confirmed via 1.0% agarose gel electrophoresis [50]. The 16S rRNA genes were amplified by PCR using the universal primer combination 27 F: 5′-AGAGTTTGATCATGGCTCAG-3′, 1492 R: 5′-TACGGTTACCTTGTTACGAC-3′ for bacteria, and 6 F: 5′-ATTCCGGTTGATCCTGCCGG-3′, 1540R: 5′-AGGAGGTGATCCAGCCGCAG-3′ primers for archaea. The standard 25 µL PCR reaction mixture contained genomic DNA template (~ 50 ng), 1X PCR buffer (1.5 mM MgCl_2_), 0.2 mM dNTPs, 0.4 µM of each primer, and 1 U of *Taq* DNA polymerase. Thermocycling conditions consisted of an initial denaturation at 95 °C for 5 min, followed by 35 cycles of 95 °C for 30 s, annealing at 50 °C for 45 s, and extension at 72 °C for 1.5 min, with a final extension at 72 °C for 10 min.

Amplicons were subjected to Sanger sequencing. Raw bidirectional chromatograms were visually inspected for quality using Chromas, and high-quality consensus sequences were assembled using BioEdit (version 7.0.9). To ensure an absolutely high-confidence base calling and prevent false taxonomic assignments, ambiguous terminal regions were strictly trimmed. This rigorous quality filtering yielded high-quality consensus sequences ranging from ~ 290 bp to ~ 1466 bp (accession numbers provided in Supplementary Table S1).

**Fig. 2:**
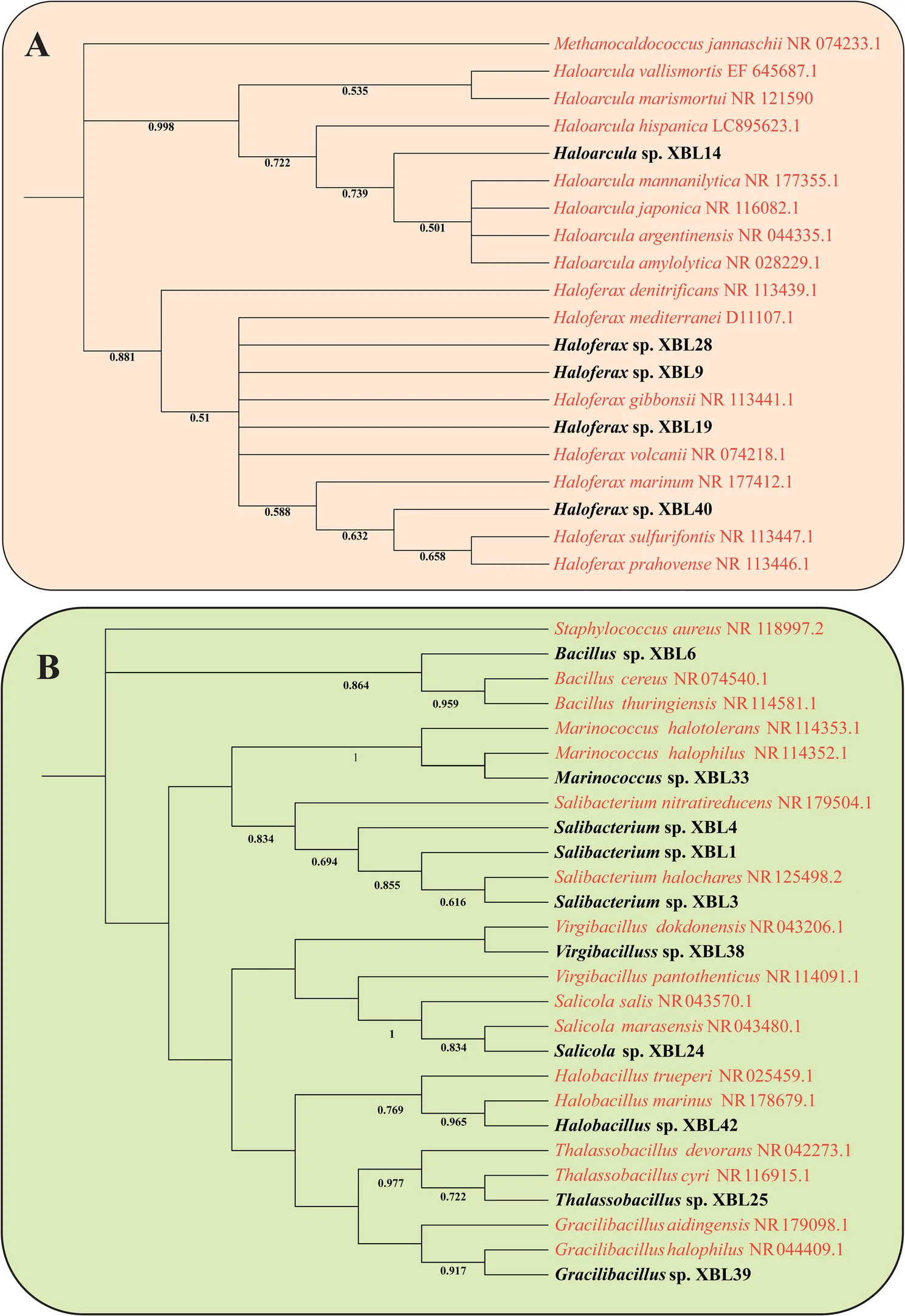
Phylogenetic reconstruction of representative halophilic isolates based on 16S rRNA gene sequences. Evolutionary relationships were inferred using the Maximum Likelihood method with bootstrap analysis based on 1000 replicates; bootstrap values > 51% are shown at the nodes. **A** Archaea isolates rooted with *Methanocaldococcus jannaschii*. **B** Bacillaceae isolates rooted with *Staphylococcus aureus*

Initial taxonomic identification was performed by comparing the consensus sequences against the NCBI GenBank database using the Basic Local Alignment Search Tool (BLASTn) and the EzBioCloud validated type strain database (http://www.ncbi.nlm.nih.gov/; https://www.ezbiocloud.net/*).* For phylogenetic reconstruction, sequences were aligned using MUSCLE within MEGA 12 [51, 52]. To ensure robust taxonomic resolution across our diverse isolate collection, a phylogenetic tree was constructed using the Maximum Likelihood (ML) method. Recognizing the distinct evolutionary dynamics of our microbial groups, independent substitution models were determined and applied: the Kimura 2-parameter model with a discrete Gamma distribution (K2 + G) was utilized for the bacterial clade (Bacillaceae). In contrast, the Tamura 3-parameter model with a discrete Gamma distribution (T92 + G) was applied to the archaeal clade (Haloferacaceae). To accurately polarize the phylogenies, the bacterial and archaeal trees were rooted using *Staphylococcus aureus* and *Methanocaldococcus jannaschii* as distant outgroups, respectively. The phylogenetic tree was evaluated via bootstrap analysis with 1,000 replicates, utilizing a ≥ 50% majority-rule consensus threshold to accommodate the high 16S rRNA sequence conservation characteristic of these extremophilic taxa (Fig. 2). Representative 16 S rRNA gene sequences were submitted to GenBank and used for phylogenetic analysis (Supplementary Table S1), and the final tree topology was visualized and annotated using the Interactive Tree Of Life (iTOL) platform [53].

### Metagenomic analysis

#### DNA extraction and quality assessment

Brine and salt from the four composite sediment–brine samples were shipped on dry ice to Unipath Specialty Laboratory Ltd. India under internal submission numbers S1, S3, S4, and S5 (corresponding to the Tamil Nadu, Rajasthan, Goa, and Gujarat samples, respectively; Project ID: 240172), where library preparation and whole-metagenome sequencing were performed on the Illumina NovaSeq X Plus platform. DNA was extracted using the DNeasy PowerSoil Pro Kit (Cat. No. ID:47014, Qiagen, Germany) following the manufacturer’s instructions. To ensure representative community capture and mitigate contamination, extractions were performed using biological replicates, and negative extraction blanks were processed in parallel. The quantity of DNA was measured with a Qubit^®^ 4.0 fluorometer (Thermo Fisher Scientific, USA). As a preliminary quality control (QC) step to verify the presence of amplifiable environmental DNA before shotgun library construction, the isolated DNA was amplified with 16S universal primer sets (8 F and 1492R) and visualised on a 1.0% (w/v) agarose gel.

#### Library preparation and sequencing

The paired-end sequencing library was prepared using the Twist NGS library preparation kits for Illumina^®^ (CAT No. /ID 104119). Hypersaline brines and crystallized sediments are characteristically low-biomass environments, so to prevent input-bias batch effects across sites, library preparation was initiated with 25 ng of input DNA, which was enzymatically sheared into smaller fragments according to the manufacturer’s protocol. This process included a combined end-repair and A-tailing step, making the DNA fragments ready for adapter ligation. Samples were multiplexed utilizing standard Illumina-specific barcoded adapters (Unique Dual Indices) to prevent barcode hopping. To maximize yield from limited input material, a high-fidelity PCR amplification step was performed using HiFi PCR Master Mix according to the manufacturer’s instructions. The amplified libraries were assessed on a TapeStation 4150 (Agilent Technologies) using High Sensitivity D1000 ScreenTape^®^, confirming a mean fragment size range from 353 to 424 bp across all samples (Supplementary Figure S1). This ensures optimal fragment size distribution and molarity.

Normalized libraries were loaded onto an Illumina NovaSeq X Plus platform for cluster generation. Sequencing was performed using a Paired-End (2 × 150 bp) configuration, generating between 4.84 and 8.68 Gb of high-quality raw data per sample.

#### Sequence assembly and gene prediction

Raw reads were quality-filtered using fastp to remove sequencing adapters and low-quality sequences. A strict filtering threshold was applied to discard bases with a Phred quality score below 20 and read fragments shorter than 36 bases. Given the environmental origin of the hypersaline samples and the robust baseline sequencing quality, additional external error correction or host-depletion algorithms were deemed unnecessary. To accurately capture localized diversity and prevent the formation of cross-site chimeric contigs, sequence data from each geographical site were assembled individually and assembled *de novo* using MEGAHIT (v1.2.9), a standalone metagenomic assembly program deliberately selected for its memory efficiency and robust capacity to resolve large, complex, and unevenly covered environmental metagenomic data. The assembled scaffolds from the sample were subjected to gene prediction using Prodigal (v2.6.3), following the metagenomic gene prediction approach (-p meta), to account for the highly diverse and anonymous nature of the environmental microbial communities [54].

#### Taxonomic annotation

These predicted genes were then further analysed for taxonomic and functional insights. Taxonomic annotation was conducted using KAIJU (v1.9.0), a protein-level classifier for metagenomic sequences [55]. The predicted protein sequences were queried against the NCBI non-redundant (nr) protein database. To maximize classification sensitivity, the analysis was run in greedy mode, allowing up to 5 amino acid mismatches. The search parameters were strictly defined with a minimum match length of 11, a minimum match score of 75, and a low-complexity sequence filter. For reads with equally good matches to multiple taxa, assignments were made utilizing the Lowest Common Ancestor (LCA) algorithm.

To evaluate community composition, the resulting taxonomic abundance data were formatted and imported into MicrobiomeAnalyst 2.0. To prevent sequencing depth bias, the data were normalized before alpha and beta diversity analyses. Additionally, to assess the metagenomic depth of coverage, rarefaction curves were generated using the vegan package in R. Community profiling and core microbiome analysis were performed at the genus level, applying a 0.01% relative abundance threshold. To ensure a comprehensive analysis of the broader pan-microbiome across all four distinct geographical sites, a 20% sample prevalence criterion (representing presence in at least one of the four sites) was deliberately applied to retain and evaluate both shared community members (core microbiome) and highly localized, site-specific unique taxa [56]. Finally, to evaluate the environmental representation of our cultured isolates, the taxonomic profiles generated from culture-independent metagenomic data were qualitatively compared with those from culture-dependent data. This parallel synthesis allowed for a comprehensive ecological overview of the extreme hypersaline microbiomes.

#### Functional annotation and screening for enzymes of biotechnological interest

To evaluate the broad metabolic potential and functional specialization of the hypersaline microbiomes, a general functional classification of whole-metagenome-predicted ORFs was performed. The sequences predicted as ORFs were subjected to functional analysis by employing the COGNIZER (v0.9b) tool on default parameters. COGNIZER is a comprehensive stand-alone framework, which uses COG, KEGG, Pfam, GO, and FIG databases to infer functional terms in the metagenomic dataset [79]. For this study’s primary comparative analysis, the multi-database output was filtered, and best-hit assignments were summarized specifically at the Clusters of Orthologous Groups (COG) functional-category level, allowing for the quantification and distribution of broad functional pathway classes across the four geographical sites. Following general functional profiling, a targeted in silico bioprospecting approach was employed to mine the metagenomes for predicting specific enzymes of high biotechnological and industrial value.

A custom reference database was constructed exclusively utilizing rigorously reviewed (Swiss-Prot) protein sequences downloaded from UniProtKB. The panel of 14 target enzymes was deliberately selected based on high commercial demand across four primary biotechnological sectors: (i) Pharmaceutical biocatalysis (Ketoreductase, Omega transaminase, Nitrillase, Monooxygenase, Peroxygenase, and Esterase) for the synthesis of chiral intermediates and active pharmaceutical ingredients (APIs); (ii) Functional food and nutraceuticals (D-allulose 3-epimerase, Cellobiose 2-epimerase, and Amylosucrase) for the bioconversion of rare sugars and prebiotics; (iii) Lignocellulosic biorefining (Cellulase, Endoglucanase, Xylanase, and Ligninase) for biomass depolymerization and biofuel generation; and (iv) Environmental bioremediation (Laccase). By targeting these specific enzyme classes, the database serves as a robust screening tool for identifying candidate novel biocatalysts with potential industrial applicability. The metagenomic ORFs were queried against this curated database utilizing MMseqs2 software suite [57]. Specifically, the core modular workflow (comprising database construction, iterative search, and alignment conversion) was executed to perform highly sensitive protein-protein sequence alignments utilizing the default BLOSUM62 substitution matrix. To isolate high-confidence candidates while accounting for the profound evolutionary divergence of extremophilic proteins, custom stringent filtering thresholds were applied. Because novel halophilic enzymes often exhibit massive sequence divergence from characterized mesophilic homologs in public databases, a baseline identity threshold of 40% was deliberately selected to maximize the discovery of distant novel variants. Structural relevance and statistical significance were strictly enforced by requiring a minimum alignment length of ≥ 100 amino acids and an e-value of ≤ 1e-5. Filtered results meeting all three independent criteria were retained for downstream analysis, effectively reducing spurious matches while preserving novel extremophilic diversity.

#### Diversity analyses

Diversity metrics were calculated using the taxonomic abundance tables at the genus level, as KAIJU (v1.9.0) performs direct read-to-reference taxonomic binning rather than traditional sequence clustering. Alpha diversity was estimated using the estimate richness of phyloseq R package [58]. Following standard best practices for asymptotic richness estimation, unrarefied raw count data were utilized. The results were reported as Chao1, ACE, Shannon, Simpson, InvSimpson, and Fisher indices. The obtained values were used to represent alpha diversity as a box plot using the plot richness function. Alpha diversity metrics are crucial for assessing the richness and evenness of microbial communities within individual samples. Richness metrics, such as Chao1 and ACE, estimate the total number of species, with a particular emphasis on rare taxa. Evenness metrics, such as Shannon and Simpson indices, provide insights into the distribution of species abundance. This study aims to explore these indices across four environmental samples to elucidate differences in microbial community structure. For beta diversity, the genus-level taxonomic abundance table derived from the KAIJU (v1.9.0) analysis was first rarified to an even sequencing depth, followed by Principal Coordinates Analysis (PCoA) and Non-metric Multidimensional Scaling (NMDS) analyses using the Bray-Curtis distance metric in the phyloseq R package. Since this study was designed as an exploratory, deep-sequencing baseline to capture the pan-microbiome of distinct, geographically isolated halophilic systems, single composite metagenomes were sequenced per macro-environment (*n* = 1 per site). Consequently, the calculation of within-group variance required for formal inferential hypothesis testing (e.g., PERMANOVA) was not feasible. Therefore, beta-diversity ordinations are presented strictly as descriptive topological representations of community differentiation, and characterized taxonomic diversity is qualitatively cross-referenced with functional enzyme profiles to establish a comprehensive baseline for these scattered extremophilic ecosystems.

#### Baseline screening of the environmental resistome and virulence factors

Potential antimicrobial resistance (AMR) and virulence genes in the metagenome were identified using ABRicate (v1.0.1). To ensure high coding-sequence specificity and eliminate false positives from non-coding structural regions, the predicted gene nucleotide sequences generated by Prodigal (rather than raw, unannotated scaffolds) were used as direct input. The sequences were queried against a comprehensive suite of established databases, including NCBI, CARD, ARG-ANNOT, Resfinder, MEGARES, and PlasmidFinder. For virulence factor screening, the general Virulence Factor Database (VFDB) was utilized. By default, ABRicate (v1.0.1) applies thresholds of 80% for both “Minimum DNA % identity” and “Minimum DNA % coverage”. Given the profound evolutionary divergence of extreme halophilic genomes from the clinical mesophilic pathogens that predominantly populate these databases, strict clinical thresholds (e.g., ≥ 90% identity) would artificially mask highly divergent, novel extremophilic variants. Therefore, the 80% thresholds were deliberately maintained to capture the broader, intrinsic environmental resistome and establish a preliminary baseline of these underexplored extremophilic habitats.

#### Secondary metabolite biosynthetic gene cluster analysis

Secondary metabolite prediction was done using antiSMASH 7.0, which accurately identifies gene clusters encoding secondary metabolites across all known major chemical classes [59]. The *de novo* assembly files generated from MEGAHIT (v1.2.9) were used as input. To ensure high-quality cluster prediction, reduce false positives from highly fragmented sequences, and adhere to computational memory limits, a strict pre-filtering size cutoff was applied i.e. only assembled scaffolds ≥ 2000 bp were retained for the antiSMASH analysis. The analysis was performed using standard default metagenomic parameters (including “Relaxed” detection strictness) to maximize detection of both complete and partially assembled BGCs inherent to environmental sequencing data. It is important to note that this screening for highly specialized secondary pharmaceutical metabolites (e.g., NRPS, PKS, bacteriocins) was conducted as a parallel, independent bioprospecting aim distinct from the primary industrial enzyme screening detailed in Section Functional Annotation and Screening for Enzymes of Biotechnological Interest.

## Results and discussion

### Physicochemical characterization of salt pan samples

Analysis of brine and salt samples from the investigated salterns provided a baseline for assessing environmental variability and its influence on microbial community composition. The pH values ranged from 6.4 to 10.2, indicating a broad gradient from near-neutral to strongly alkaline conditions. Salinity measurements reflected the distinct matrices of the sampling sites. Among the solid matrices evaluated via 1:100 aqueous extracts, the crystalline salt from Gujarat exhibited the highest electrical conductivity (9.78 ± 1.1 mS/cm), followed by the Goa sediment (8.95 ± 0.8 mS/cm) and the highly alkaline Rajasthan sediment (1.60 ± 0.3 mS/cm). The Tamil Nadu liquid brine, measured directly, recorded an electrical conductivity of 25.0 ± 1.5 mS/cm. Accordingly, quantitative comparative interpretations of salinity were restricted to intra-matrix evaluations. Temperature measurements recorded during sampling ranged narrowly (34 to 43 **°**C) across sites, consistent with ambient field conditions, and are unlikely to represent a primary driver of community differentiation compared to salinity and ionic composition (Table 1).

**Table 1:** Sample collection sites and their physiological parameters and characteristics. The table lists sample data (pH, EC, and Temperature) of the brine, soil and salt samples from Indian halophilic sites

Location	Sample Type	pH	Direct EC (mS/cm)	Extract EC₁:₁₀₀ (mS/cm)	Temp. (°C)
Naravakkam, Tamil Nadu	Water (brine)	7.7 ± 0.1	25.0 ± 1.5	-	36 ± 2
Sambhar Salt Lake, Rajasthan	Soil	10.2 ± 0.1	-	1.60 ± 0.3	43 ± 1
Betim Salt Pan, Goa	Soil	8.3 ± 0.1	-	8.95 ± 0.8	34 ± 2
Bhimrana, Gujarat	Salt	6.4 ± 0.1	-	9.78 ± 1.1	42 ± 2

### Identification and phylogenetic analysis of culturable strains

#### Morphological and biochemical observations

A total of 42 morphologically distinct isolates were obtained by repeated streaking of the collected salt and brine samples on EHM and MHM media. Colonies exhibited variations in pigmentation, texture, and growth rate, suggesting the presence of diverse halophilic taxa. Genera such as *Haloferax* and *Haloarcula* displayed strong red-to-orange colors, which are due to C_50_ carotenoids such as bacterioruberin. These pigments help protect the cells from light and keep them stable in salty conditions [60]. An entire margin with a circular colony was observed in most isolates. Gram-staining and biochemical assays differentiated the isolates into Gram-positive and Gram-negative rods, with several displaying pigment production (orange, red, yellow) typical of halophilic archaea and bacteria (Supplementary Table S1). The enzymatic profile of these halophilic (salt-loving) isolates revealed strong esterase and amylase activity, with the highest clearance zones of 9.0 ± 1.25 mm and 9.0 ± 1.36 mm, respectively (Supplementary Table S2). The presence of these enzymes indicates that these isolates are well-suited to breaking down proteins, lipids, and carbohydrates under stressful environments. This suggests these extremophiles have evolved to break down algal and brine shrimp biomass commonly found in such halophilic environments. These halostable enzymes can be important for nutrient cycling and hold potential in white biotechnology applications, such as saline wastewater treatment, thereby highlighting the need for further kinetic characterization [61–63]. Our culture-based results support and add to the findings of Das et al. [38], showing that these multi-enzyme producers are key decomposers in Indian hypersaline ecosystems. In contrast, reduced activity of xylanase and cellulase suggests a decreased capacity to digest complex plant-derived lignocellulose. Despite the extreme osmotic stress in their natural habitats, a considerable proportion (31% i.e., 13 of the 42 isolates) exhibited multi-enzymatic adaptability, secreting three unique types of hydrolases.

#### 16S rRNA gene sequencing and identification

All 42 isolates showing distinct phenotypic characteristics were subjected to genomic DNA extraction using the CTAB method, followed by amplification and Sanger sequencing of the 16S rRNA gene. Following rigorous quality trimming of ambiguous ends, high-confidence consensus sequences ranging from ~ 290 bp to ~ 1466 were generated. The resulting sequences were compared with reference sequences using BLASTn searches against the NCBI database. The complete taxonomic classification, along with the molecular characteristics of the 42 isolated microbial strains, is presented in Supplementary Table S1. To prevent visual redundancy, representative non-redundant sequences were selected from these groups for phylogenetic reconstruction (Fig. 2). While these strictly quality-controlled, partial (< 1200 bp) sequences, combined with the EzBioCloudvalidated typestrain database, adequately cover key hypervariable regions to provide highly robust genus-level resolution, we acknowledge that absolute species-level delineation within highly conserved clades (such as Bacillaceae) may require future whole-genome sequencing.

A diverse group of organisms, representing both archaea and bacteria, was recovered. The isolates exhibited 93–100% sequence similarity to known taxa belonging to the phyla Firmicutes, Proteobacteria, Actinobacteria, and Euryarchaeota. Notably, isolates exhibiting sequence similarities near the 93% lower bound strongly suggest the discovery of novel halophilic taxa; however, formal taxonomic propositions requiring exhaustive polyphasic characterization remain outside the primary ecological scope of this study. Archaea isolates formed well-supported clusters within the class Halobacteria, with *Haloarcula* and *Haloferax* resolving as distinct lineages. XBL14 formed a distinct cluster with *Haloarcula*, while XBL28, XBL9, XBL19, and XBL40 formed a distinct cluster with *Haloferax*. *Methanocaldococcus jannaschii* was used as the outgroup for archaeal phylogenetic reconstruction. Within the bacterial domain, a clear clade affiliated with the family Bacillaceae was resolved. Isolate XBL6 clustered with *Bacillus cereus*, while isolates XBL1, XBL3, and XBL4 formed a distinct cluster with *Salibacterium halochares*. XBL33, XBL38, XBL24, XBL42, XBL25, and XBL39 aligned with *Marinococcus*,* Virgibacillus*,* Salicola*,* Halobacillus*,* Thalassobacillus*, and *Gracilibacillus*, respectively. *Staphylococcus aureus* was used as the outgroup to anchor this bacterial lineage.

Among bacteria (15), members of the phylum Firmicutes were predominant (14 out of 15), including representatives of *Salibacterium* (3), *Halobacillus* (3), *Marinococcus* (3), *Gracilibacillus* (2), *Bacillus* (1), *Thalassobacillus* (1), and *Virgibacillus* (1), alongside a single *Salicola* isolate. Archaea (27) were dominated by *Haloarcula and Haloferax*, with *Haloarcula* emerging as the most abundant archaeal genus, comprising 18 isolated strains. Many of the identified taxa are known for their relevance in biotechnological applications, industrial enzyme production, and environmental sustainability [9, 64].

#### Phylogenetic analysis

Phylogenetic analysis was performed to examine the evolutionary relationships among the cultured isolates and to assess their affiliation within established halophilic lineages. The Maximum Likelihood tree (1,000 bootstrap replicates; ≥51% support shown) revealed well-resolved clustering patterns that were largely consistent with taxonomic assignments based on 16S rRNA gene similarity, while also highlighting intra-genus diversity among several groups. Archaea isolates formed strongly supported clades within the class Halobacteria, with *Haloarcula* and *Haloferax* resolving as distinct and coherent lineages supported by high bootstrap values (> 90%). The use of *Methanocaldococcus jannaschii* as a distant outgroup provided clear rooting of the archaeal branches.

Within the bacterial domain, isolates affiliated with the family Bacillaceae clustered into multiple subclades rather than forming a single homogeneous group, indicating phylogenetic heterogeneity among halophilic Firmicutes recovered from the saltern environments. Notably, certain isolates occupied discrete positions within their respective clades, suggesting diversification at the strain level despite high overall sequence similarity. These patterns are consistent with adaptive radiation under hypersaline conditions and may reflect localized selection pressures across sampling sites. Overall, the phylogenetic structure depicted in Fig. 2 supports the presence of multiple halophilic lineages coexisting within Indian salterns and provides a culture-based framework that complements the metagenomic evidence for archaeal and bacterial diversity under hypersaline stress.

### Metagenome sequencing analysis

#### Sequence assembly

Metagenomic sequencing produced assemblies of variable depth and complexity across the four sampled regions. Although all datasets exhibited the expected signatures of hypersaline microbiomes, including high genomic diversity and a broad distribution of contig lengths, the assemblies differed markedly among sites Table 2, Supplementary Table S3. The Gujarat sample yielded the largest dataset, recording the highest total raw data (8.68 Gb), total assembled bases (380.9 Mb), and total predicted genes (669,991). It also demonstrated the most contiguous assembly, with a Scaffold N50 of 1,279 bp. The Rajasthan sample presented intermediate metrics, with a total assembled bases of 283.5 Mb and a coding density of 87.6%.

**Table 2:** Summary of genome sequencing, assembly, and annotation statistics

Region	Total Raw Data (Gb)	Total Assembled Bases (Mb)	Scaffold N50 (bp)	Max Scaffold Size (bp)	Total Predicted Genes	Coding Density (%)
Tamil Nadu	4.84	281.8	903	65,322	503,796	89.2
Rajasthan	6.22	283.5	953	335,560	517,927	87.6
Goa	5.32	233.3	1,025	416,255	429,420	88.7
Gujarat	8.68	380.9	1,279	383,639	669,991	87.0

*A scaffold is a set of contiguous DNA sequences (or contigs) that have been ordered and oriented. N50 is defined as the sequence length at which 50% of the total assembled bases are contained in scaffolds of that length or longer. Coding Density = (Total length of coding sequence (bp) / Total assembled bases (bp)) * 100

Overall, the assembly statistics reflect the profound taxonomic and structural complexity inherent to extreme environmental microbiomes. The highly fragmented nature of these assemblies (with N50 values ranging from 903 to 1,279 bp) is a well-documented technical hallmark of complex sediment-like metagenomes. This fragmentation is primarily driven by massive intra-site strain heterogeneity, which intrinsically limits the capacity of de Bruijn graph assemblers to generate long contiguous scaffolds.

This biological complexity is further reflected in the maximum scaffold variations. In contrast to Gujarat, the Tamil Nadu sample generated the lowest raw data (4.84 Gb) and had the smallest maximum scaffold size (65,322 bp); however, it exhibited the highest coding density at 89.2%. The Goa sample, despite having the smallest total genome length (233.3 Mb) and lowest gene count (429,420), achieved the largest maximum scaffold size of 416,255 bp. This dramatic 6.4-fold difference in maximum scaffold length likely reflects differences in localized biological dominance i.e. environments heavily dominated by a few clonal extremophilic populations (e.g., Goa) permit the assembly of longer, contiguous genomic islands, whereas highly even, hyper-diverse communities (e.g., Tamil Nadu) result in more entangled assembly graphs.

Despite this expected fragmentation, the assemblies yielded a massive repository of functional potential. While the Tamil Nadu sample exhibited the highest raw coding density at 89.2% (with Rajasthan at 87.6%), these elevated values (87.0–89.2% across all sites) are characteristic of Prodigal’s metagenomic mode, which aggressively predicts partial open reading frames (ORFs) on the edges of short contigs. However, the subsequent downstream application of strict size and identity filters (e.g., the ≥ 100 amino acid threshold detailed in Section Functional Annotation and Screening for Enzymes of Biotechnological Interest) successfully eliminated these partial false-positive artifacts before our targeted bioprospecting analyses. 

#### Microbial taxonomy annotation

To assess the metagenomic depth of coverage across the sampling sites, rarefaction analysis was performed using the taxonomic abundance matrix. The sequencing depth yielded between ~ 240,000 and 350,000 high-quality reads per sample. The resulting rarefaction curves for all geographic sites exhibited a clear plateauing trend (Supplementary Figure S2). This transition to a shallow slope demonstrates diminishing returns, indicating that the sequencing depth was robust and sufficient to capture the vast majority of the microbial diversity, including the core community, present within these hypersaline environments.

**Fig. 3:**
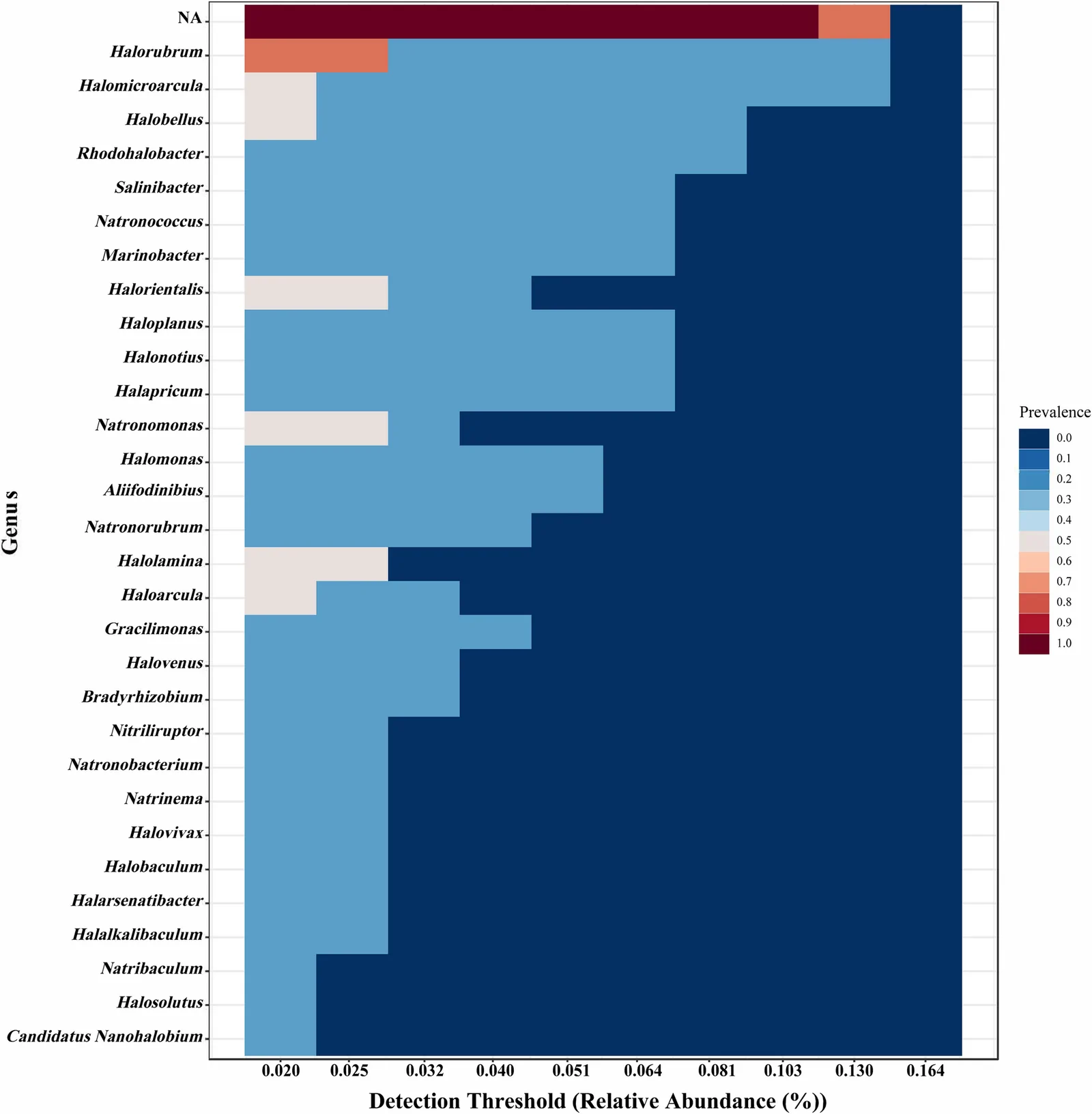
Core prevalence of dominant microbial genera across the sampled hypersaline environments. Taxonomic classification was performed using KAIJU (v1.9.0). The visualization includes only genera that passed a 0.01% abundance threshold and appeared in at least 20% of the samples. The colour legend corresponds to the major genera shared across the sampling sites

Taxonomic annotation using KAIJU (v1.9.0) revealed pronounced differences in microbial community composition across the sampled salterns. Archaeal sequences were predominant in samples from Gujarat, Goa, and Rajasthan, whereas comparatively lower archaeal representation was observed in the Tamil Nadu sample (Supplementary Figure S3 and Supplementary Figure S4). A substantial fraction of the dataset remained unclassified at the genus level, reflecting the presence of poorly characterized or novel taxa typical of hypersaline environments. Among the classified genera, *Halorubrum* showed the highest relative abundance and core prevalence across the sampled sites, followed by *Halomicroarcula*,* Haloarcula*, and *Halobellus* (Fig. 3).

Comparison across sites demonstrated distinct taxonomic profiles associated with local environmental conditions. High-salinity samples were dominated by halophilic archaeal genera, whereas samples from lower salinity conditions exhibited increased bacterial representation. Overall, the community structure displayed a strongly skewed distribution, characterized by a small number of highly abundant genera and a large pool of low-abundance taxa. This long-tail pattern suggests strong environmental filtering and niche specialization within the saltern microbiomes.

The taxonomic patterns observed in Fig. 3 provide a metagenome-level overview of community organization across the investigated sites and establish a foundation for subsequent diversity, functional, and enzyme-centric analyses. These results also complement the culture-dependent phylogenetic findings, collectively highlighting the dominance of archaeal lineages in hypersaline conditions and the coexistence of diverse bacterial taxa under less extreme regimes.

**Fig. 4:**
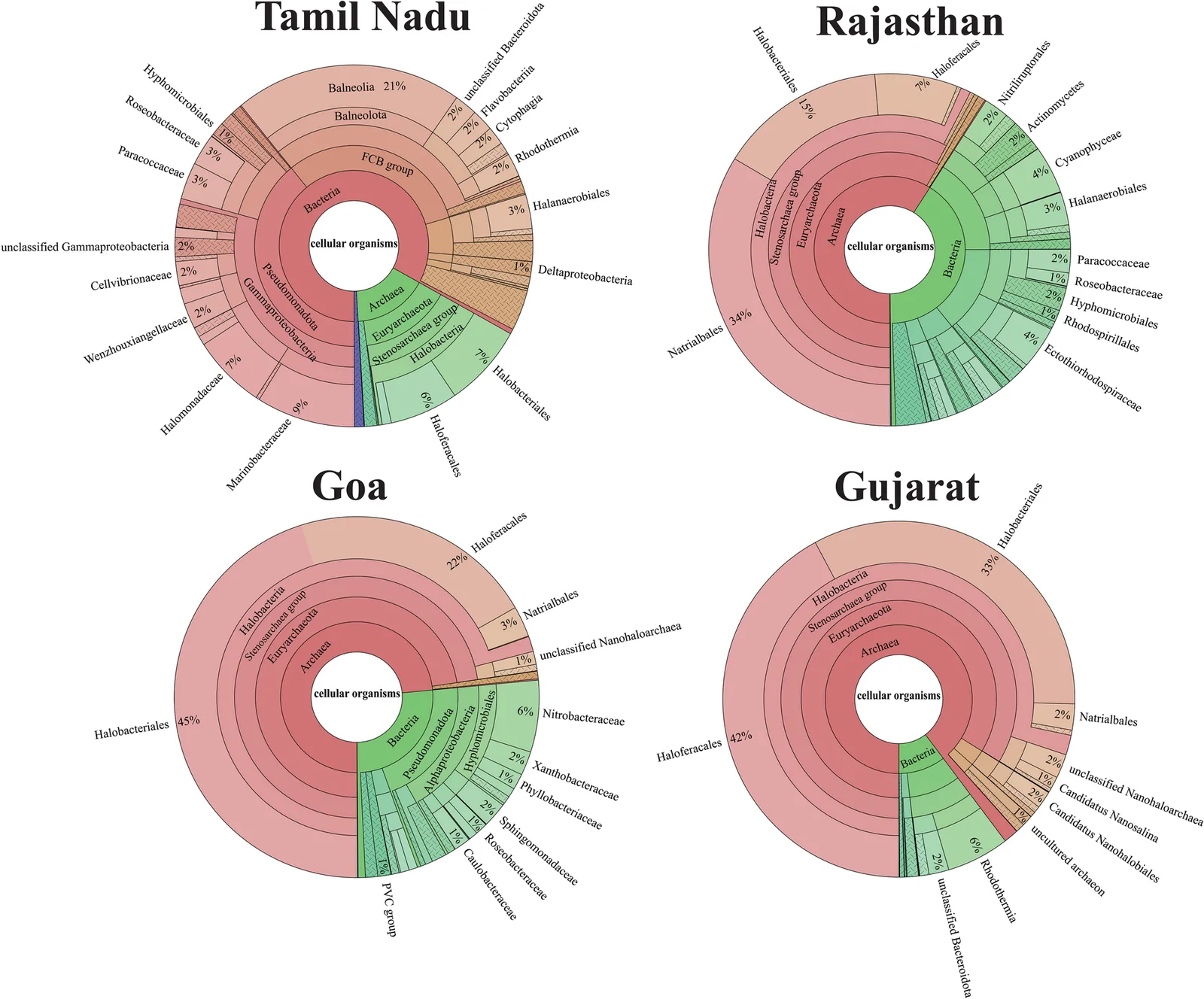
Krona plots illustrating taxonomic composition across hypersaline sites. The Krona plot was assessed up to taxonomic depth 7, thus representing the major taxonomic orders detected in the samples. Each plot displays a hierarchical representation of taxa identified by KAIJU (v1.9.0)

The hierarchical taxonomic structure of the microbial communities was visualized using Krona plots (Fig. 4). The comparative community composition across the four sites is summarized in Table 3, Supplementary Table S4. In Tamil Nadu, the microbiome was predominantly bacterial (83%), with major groups comprising Bacteroidota (formerly Bacteroidetes)/Chlorobiota (30%), Gammaproteobacteria (29%), and Alphaproteobacteria (9%). Among Gammaproteobacteria, Pseudomonadales (31%) and Oceanospirillales (8%) were the key contributors. Bacteroidota (7%) play a significant role in the degradation of organic matter. At the genus level, the community was characterized by the highest relative evenness, led by *Marinobacter* (5.6%) and *Rhodohalobacter* (5.1%). Archaea comprised 16% of the microbiome, mainly represented by Halobacteria within Euryarchaeota (15%). Eukaryotes were present in a small proportion (0.9%), suggesting a limited ecological contribution. Balneolota was primarily found, suggesting a preference for moderately saline or fluctuating conditions. This observation is consistent with reports from hypersaline freshwater systems such as the Malaya Samoroda River and an associated pond, where Balneolota populations were linked to intermediate salinity and organic matter availability [65].

**Table 3:** Summary of the dominant microbial community composition across the four investigated saltern sites

Geographic Site	Dominant Domain (%)	Major Phyla/Classes (%)	Top Abundant Genera (%)
Tamil Nadu	Bacteria (83%)	Bacteroidota (30%), Gammaproteobacteria (29%)	*Marinobacter* (5.6%), *Rhodohalobacter* (5.1%)
Rajasthan	Archaea (59%)	Halobacteria (57%), Pseudomonadota (14%)	*Natronococcus* (3.7%), *Natronorubrum* (2.4%)
Goa	Archaea (73%)	Halobacteria (71%), Pseudomonadota (21%)	*Halomicroarcula* (10.4%), *Bradyrhizobium* (4.2%)
Gujarat	Archaea (90%)	Halobacteria (80%), Bacteroidota (9%)	*Halorubrum* (12.6%), *Halobellus* (4.9%)

Rajasthan exhibited a notable presence of Archaea (59%), predominantly Euryarchaeota (58%), mainly in the form of Halobacteria (57%). At the genus level, *Natronococcus* (3.7%) and *Natronorubrum* (2.4%) were the most prominent. Bacteria comprised 41% of the community, with a diverse range including Pseudomonadota (formerly Proteobacteria) (14%), Alphaproteobacteria (7%), Actinomycetota (formerly Actinobacteria) (6%), and Cyanobacteriota (formerly Cyanobacteria) (4%). Actinomycetota (6%) were notable for their potential to produce bioactive compounds. Eukaryotes were negligible, contributing < 0.1% and having minimal ecological relevance.

In Goa, Archaea were dominant (73%), with the majority being Euryarchaeota (73%) and Halobacteria (71%) as the major class. Haloferacales (22%) were highly abundant, thriving in extreme salinity conditions. The genus *Halomicroarcula* was exceptionally dominant at this site, accounting for 10.4% of the classified community, followed by *Bradyrhizobium* (4.2%) and *Haloplanus* (4.1%). Nanohaloarchaea (2%) were also present, indicating the presence of potential novel extremophiles. Bacteria constituted 26% of the microbiome, including Pseudomonadota (21%), Alphaproteobacteria (18%), and Gammaproteobacteria (2%). Eukaryotes were present at a very low level (0.08%), contributing minimally to the community. The occurrence of Cyanobacteria in Rajasthan and Verrucomicrobiota in Goa illustrates strong regional differentiation in community composition. Such patterns likely reflect site-specific combinations of salinity, water influx, nutrient availability, and climatic conditions rather than salinity alone.

In Gujarat, Archaea accounted for upto 90% of the microbiome, with the majority being Euryarchaeota (84%) and mainly Halobacteria (80%). Haloferacales (42%) were the most abundant, adapted to high-salt environments. At the genus level, *Halorubrum* was the undisputed dominant taxa, reaching its highest relative abundance (12.6%) across all samples, followed by *Halobellus* (4.9%). Bacteria made up 10% of the community, including Alphaproteobacteria (1%), Bacteroidota (9%), and Rhodothermota (6%). Eukaryotes were nearly absent, with only 0.02% present, contributing minimally. Unclassified and novel groups, including Nanohaloarchaea, suggested the presence of previously uncharacterized extremophilic lineages. The detection of *Candidatus* Nanohaloarchaeota exclusively in Gujarat points to niche specialization under extreme ionic stress. Members of the DPANN superphyla, have previously been reported from highly saline deserts and crystallizer ponds globally, including the Atacama Deserts and Mediterranean solar salterns [66–68] and regionally in the hypersaline Sambhar Salt Lake [69], suggesting that Gujarat salterns represent a similarly selective ecological niche. Moreover, their presence suggests that these enigmatic, interdependent communities possess a broader geographic distribution across India’s extreme hypersaline ecosystems than previously recognized and may serve as persistent indicators of high-salinity ecological climax.

At the taxonomic level of the phyla and species, the yellow-to-blue and blue-to-red gradient in the heatmap highlights location-specific microbial dominance, with higher abundances in Gujarat and Rajasthan (Supplementary Figure S3 and Supplementary Figure S4). These findings highlight how salinity, climate, and regional ecological factors drive microbial community structure, providing insights into the environmental adaptability of different microbial taxa. Similar dominance of Pseudomonadota were reported from other extreme systems, such as Puga geothermal geyser in Ladakh, where 67.14% of bacterial sequences belonged to this phylum and 2.84% of archaea to Euryarchaeota [70]. The enrichment of Euryarchaeota, particularly halophilic lineages, reflects their superior physiological capacity to withstand osmotic stress through specialized ion pumps, compatible solute strategies, and protein adaptations. Both bacteria and archaea were consistently detected, with halophilic archaeal genera such as *Halorubrum* and *Haloarcula* dominating high-salinity sites. The consistent detection of Halobacteriaceae across Gujarat and Rajasthan highlights the strong association between persistent hypersalinity and archaeal-dominated communities. In contrast, Goa exhibited reduced taxonomic diversity, likely influenced by estuarine mixing and periodic freshwater input, while Tamil Nadu supported a more even assemblage of halophilic and non-halophilic taxa. The observed clustering patterns collectively indicate that microbial community assembly in Indian salterns is driven by a combination of salinity intensity, climate regime, and geographic isolation, leading to distinct ecological selection pressures across regions. For instance, among the solid matrices, the high electrical conductivity observed in Gujarat (9.78 mS/cm, 1:100 extract) aligned with the prominent recovery of Halobacteriaceae, indicating a strong association between salinity intensity and microbial community composition. Separately, the liquid brine from Tamil Nadu (direct EC of 25.0 mS/cm) exhibited a distinct physicochemical and taxonomic profile, with a relatively reduced dominance of extreme halophiles. The observed separation of clusters further supports the hypothesized selective pressure exerted by environmental extremes, particularly salinity and matrix type, on microbial community assembly.

#### Diversity analysis

**Fig. 5:**
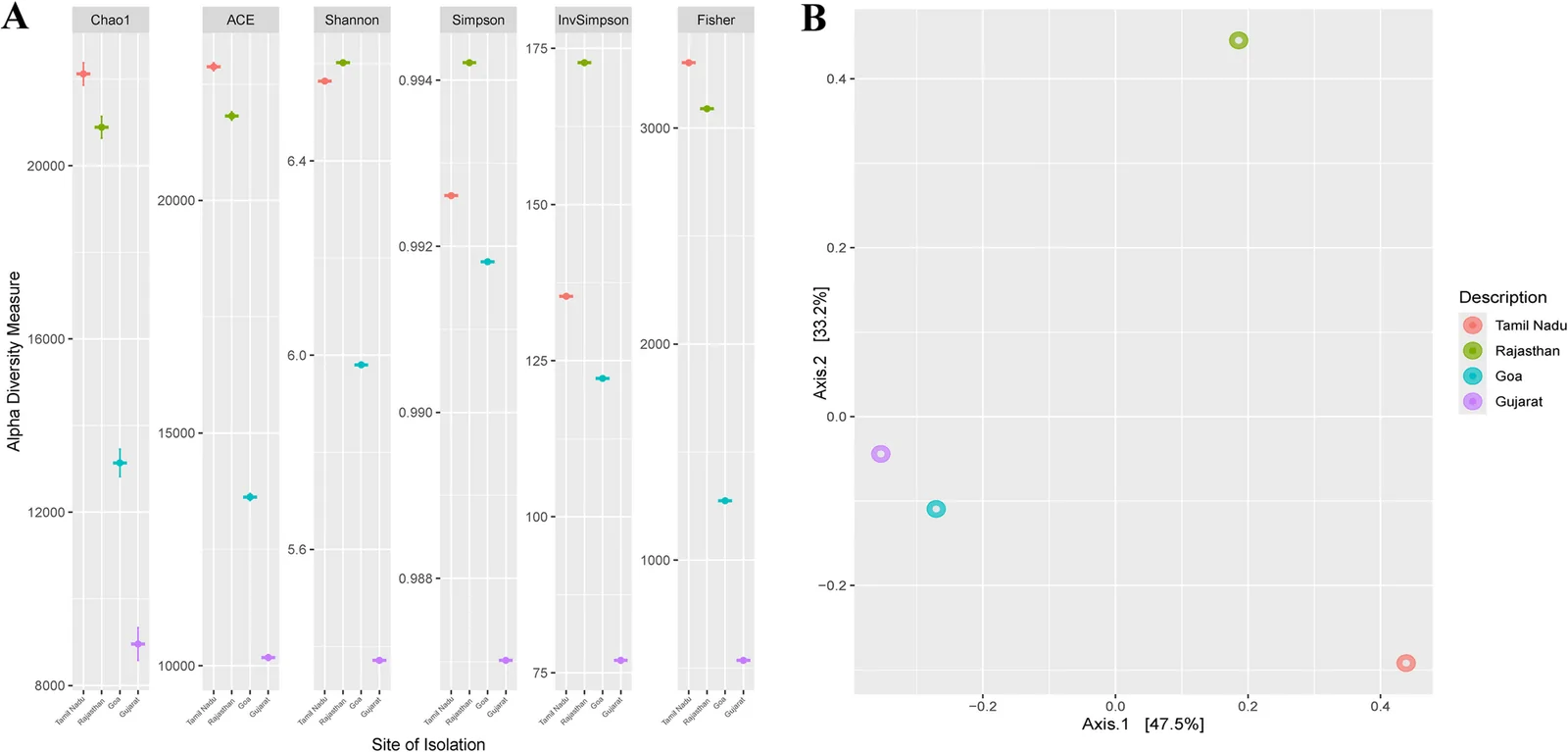
**A** Alpha diversity indices for microbial communities across different samples. Indices include Chao1 and ACE (abundance-based coverage), Shannon and Simpson (diversity dominance), Inverse Simpson (evenness), and Fisher’s alpha (parametric diversity). Each point represents a sample, with error bars indicating confidence intervals or standard errors. Different colours represent distinct sample groups. **B**: Principal Coordinate Analysis (PCoA) of microbial communities based on Bray–Curtis dissimilarity. Each point represents a sample, with colours indicating geographical locations. Distances between points reflect differences community composition

The alpha diversity analysis highlights significant differences in microbial community structure across the four environmental samples (Fig. 5A). Species richness, assessed through Chao1 and ACE indices, revealed that samples from Tamil Nadu and Rajasthan exhibited the highest richness, indicating a diverse array of microbial taxa. Conversely, samples from Gujarat showed lower richness, suggesting fewer unique taxa.

Species evenness and balance were evaluated using Shannon, Simpson, and Inverse Simpson indices, demonstrating that Rajasthan exhibited the most even species distribution (Shannon: 6.60; InvSimpson: 172.7). In contrast, Gujarat displayed the lowest evenness and highest dominance (InvSimpson: 77.0), indicating that specific taxa likely dominate this environment. Species distribution, measured using Fisher’s Alpha, further supported the high biodiversity of Tamil Nadu (Fisher: 3302.4) and Rajasthan, while Gujarat showed the lowest value (Fisher: 535.7).

These diversity patterns reflect strong environmental control over community structure. The high diversity observed in Tamil Nadu and Rajasthan suggests these environments are biologically complex and nutrient-rich, support a large reservoir of low-abundance taxa. Meanwhile, the limited diversity in Gujarat is consistent with strong environmental filtering under extreme hypersaline conditions that favour a limited number of highly adapted specialists. Together, these alpha diversity metrics provide quantitative support for site-specific ecological differentiation across the salterns.

The Principal Coordinates Analysis (PCoA) results revealed clear separation of microbial communities across Tamil Nadu, Rajasthan, Goa, and Gujarat, likely influenced by environmental gradients such as salinity and freshwater availability (Fig. 5B). The first two principal coordinates (Axis 1 and Axis 2) explained a cumulative 80.7% of the total variance (47.5% and 33.2%, respectively). The Sambhar Salt Lake (Rajasthan) sample was highly distinct, separating strongly along Axis 2 (+ 0.445). The Goa and Gujarat salt pan samples clustered closely in the lower-left quadrant (Axis 1 values of -0.270 and − 0.354, respectively), indicating a high degree of compositional similarity. In contrast, the Tamil Nadu salt pan sample separated along the positive side of Axis 1 (+ 0.439) while remaining negatively positioned on Axis 2 (-0.292), highlighting pronounced regional differentiation. These beta diversity patterns demonstrate that microbial community composition is structured by geography and local environmental conditions, with hypersaline stress acting as one of the key driver of community divergence across sites.

#### Functional annotation

**Fig. 6:**
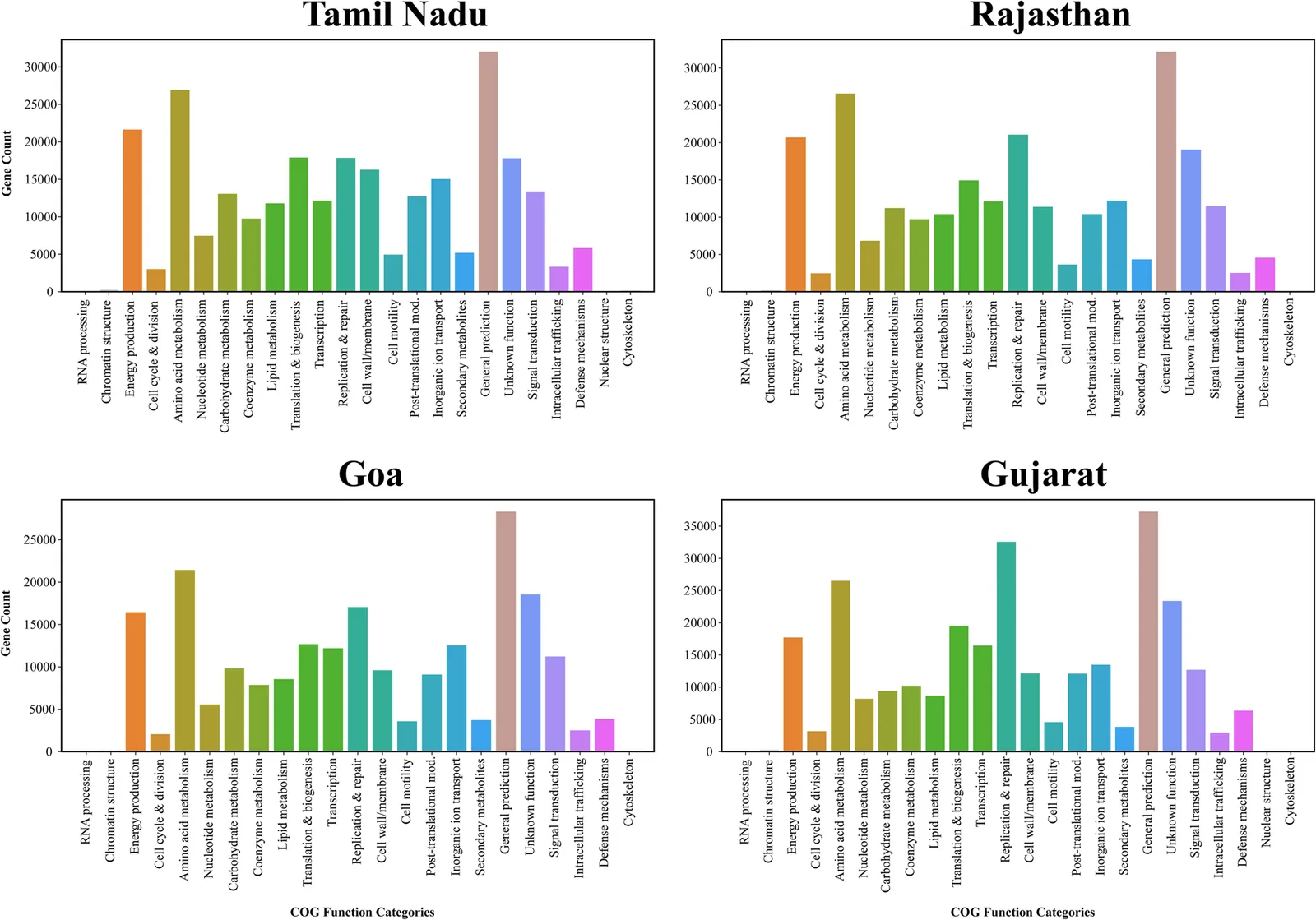
Number of genes annotated to different pathways across the samples. Annotated genes were classified using the COG database, and their distribution among functional classes is shown. Gene abundance is represented numerically, with varying bar height indicating relative abundance

The microbial communities in Tamil Nadu, Rajasthan, Goa, and Gujarat were characterized by diverse functional categories based on genomic data (Fig. 6, Supplementary Table S5). Across all sites, metagenomic functional annotation revealed that energy-intensive ion-exchange homeostasis is a central survival strategy for hypersaline microbial communities. The enrichment of genes involved in energy production and conversion across all regions indicates a substantial metabolic investment required to maintain cellular stability under high osmotic stress.

Many genes related to energy production and conversion were consistently detected in salt pan and lake samples from Tamil Nadu, Goa, Gujarat, and Rajasthan, suggesting that these microbes expend considerable energy to counter osmotic stress through active ion transport and osmolyte synthesis. The high abundance of genes associated with inorganic ion transport and metabolism (e.g., 13,478 in Gujarat and 12,199 in Rajasthan) support this interpretation, as cells must continuously regulate intracellular ionic balance by transporting sodium, potassium, and chloride via specialized transport systems. This observation is consistent with earlier studies reporting enrichment of ion transport operons, including mnhB-E and atpA-F, I, K in hypersaline soils and sediments [71, 72].

Genes related to amino acid transport and metabolism (e.g., 26,891 in Tamil Nadu, 26,506 in Gujarat, and 26,564 in Rajasthan) further indicate the synthesis and accumulation of compatible solutes such as glycine, betaine, ectoine, and proline, which are well-established osmoprotectants in halophiles. These compounds stabilize proteins and enzymatic systems under high salinity, thereby preserving metabolic activity [73, 74]. Genes involved in lipid transport and metabolism are also prominent (11,792 in Tamil Nadu, 10,403 in Rajasthan), likely reflecting membrane remodeling processes that enhance membrane rigidity and functionality under elevated salt concentrations.

Prolonged exposure to hypersaline conditions imposes oxidative and genotoxic stress, which is reflected in the enrichment of genes involved in replication, recombination, and DNA repair (32,554 in Gujarat and 21,059 in Rajasthan). Halophilic microorganisms are known to accumulate carotenoids and other antioxidants molecules that mitigate damage caused by reactive oxygen species generated under high salinity and intense solar radiation [75]. These features underscore the value of halophiles as model systems for understanding stress tolerance mechanisms and for developing stress-resilient enzymes and biomolecules.

Overall, these results highlight site-specific functional differentiation among the microbial communities, with several functional categories showing regional enrichment, while others remain poorly characterized and warrant further investigation.

**Fig. 7:**
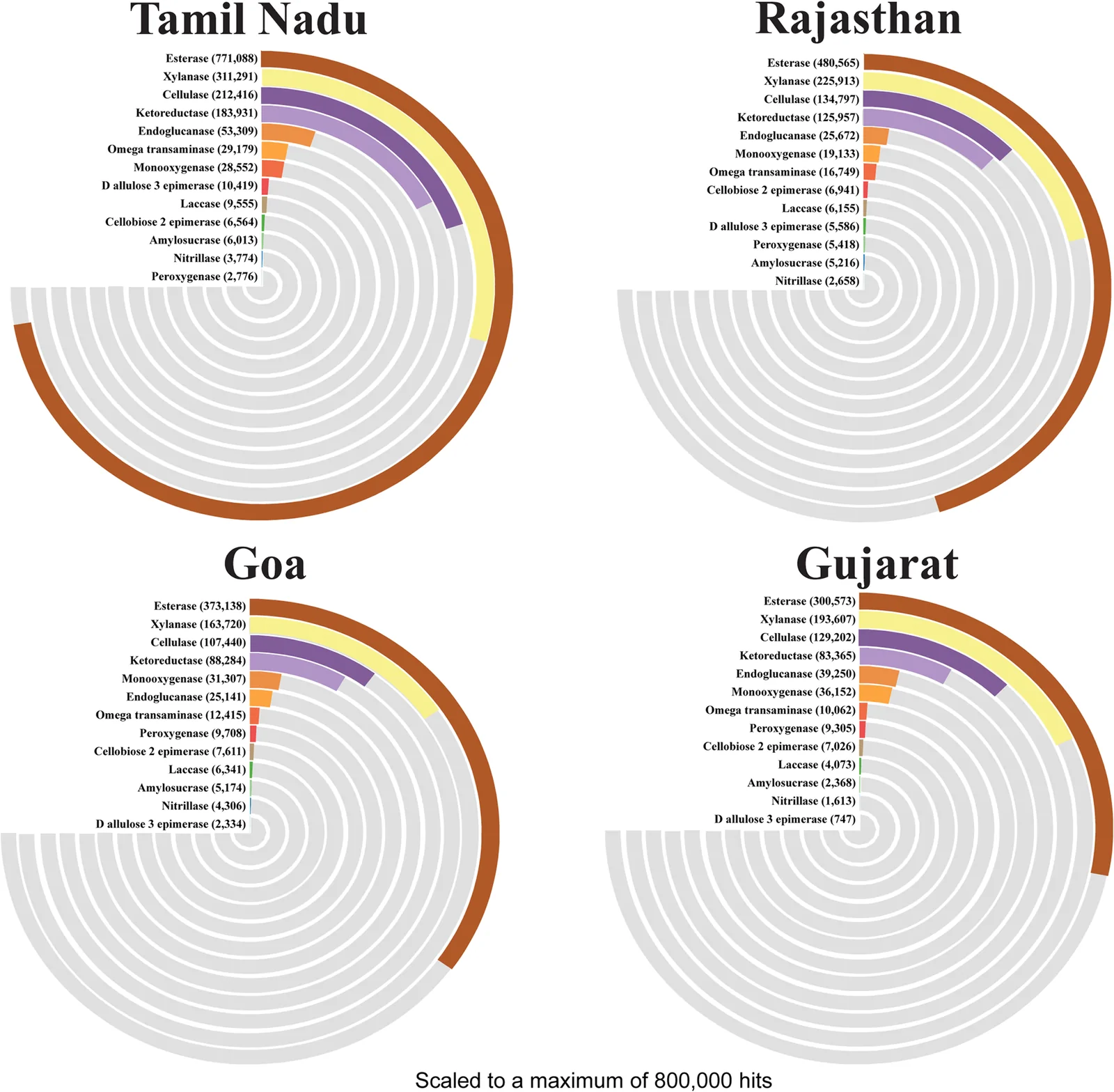
Sample-wise enzyme abundance profiles from metagenomic analysis. The plots illustrate the relative abundance of key enzyme classes across Tamil Nadu, Rajasthan, Goa, and Gujarat. Each enzyme class is represented by a concentric ring, with arc length corresponding to the number of hits, normalized to a maximum of 800,000

Metagenomic prediction followed by MMseq2-based functional annotation identified filtered, high confidence hits corresponding to 14 enzyme classes across the samples. After filtering, the number of hits was substantially reduced, and the retained sequences represented putative enzyme-coding genes with high-confidence. The number of retained hits is shown in Fig. 7.

#### Detection of antimicrobial resistance and virulence genes

The virulence factor and antibiotic resistance gene (ARG) profiles of samples are represented in Table 4. The antimicrobial resistance (AMR) gene analysis revealed that ARGs were restricted exclusively to the Goa saltern. Goa displayed a diverse but low-abundance resistance profile, with a total of 12 ARG hits detected across multiple databases, including MEGARES (4), CARD (3), NCBI (2), ARG-ANNOT (2), and ResFinder (1). In contrast, Tamil Nadu, Rajasthan, and Gujarat showed no detectable antimicrobial resistance genes. Conversely, virulence factors were more widely distributed across the geographic sites. Using the VFDB database, 14 virulence-associated genes were detected in Goa, 11 in Tamil Nadu, and 1 in Rajasthan. Gujarat remained devoid of any detectable virulence or resistance signatures.

**Table 4:** Virulence factor and antibiotic resistance gene (ARG) profiles across saltern samples. The table shows the number of hits detected in the samples for each resistance and virulence database (ARG-ANNOT, CARD, MEGARES, NCBI, ResFinder, and VFDB) for each sample

Virulence Factor (VF) profile					
Database	Tamil Nadu	Rajasthan	Goa	Gujarat	Total Hits
VFDB	11	1	14	0	26

Antibiotic Resistance Gene (ARG) profile					
Database	Tamil Nadu	Rajasthan	Goa	Gujarat	Total Hits
CARD	0	0	3	0	3
MEGARES	0	0	4	0	4
NCBI	0	0	2	0	2
ARG-ANNOT	0	0	2	0	2
ResFinder	0	0	1	0	1

Overall, these results indicate that resistance-associated genes were unevenly distributed across sites, with Goa exhibiting the highest relative abundance, whereas the remaining salterns showed minimal or no detectable AMR signatures. Although minimal but the presence of resistance determinants indicates that adaptive and gene exchange processes can operate even in extreme environments [76].

#### Secondary metabolite biosynthetic gene cluster analysis

**Fig. 8:**
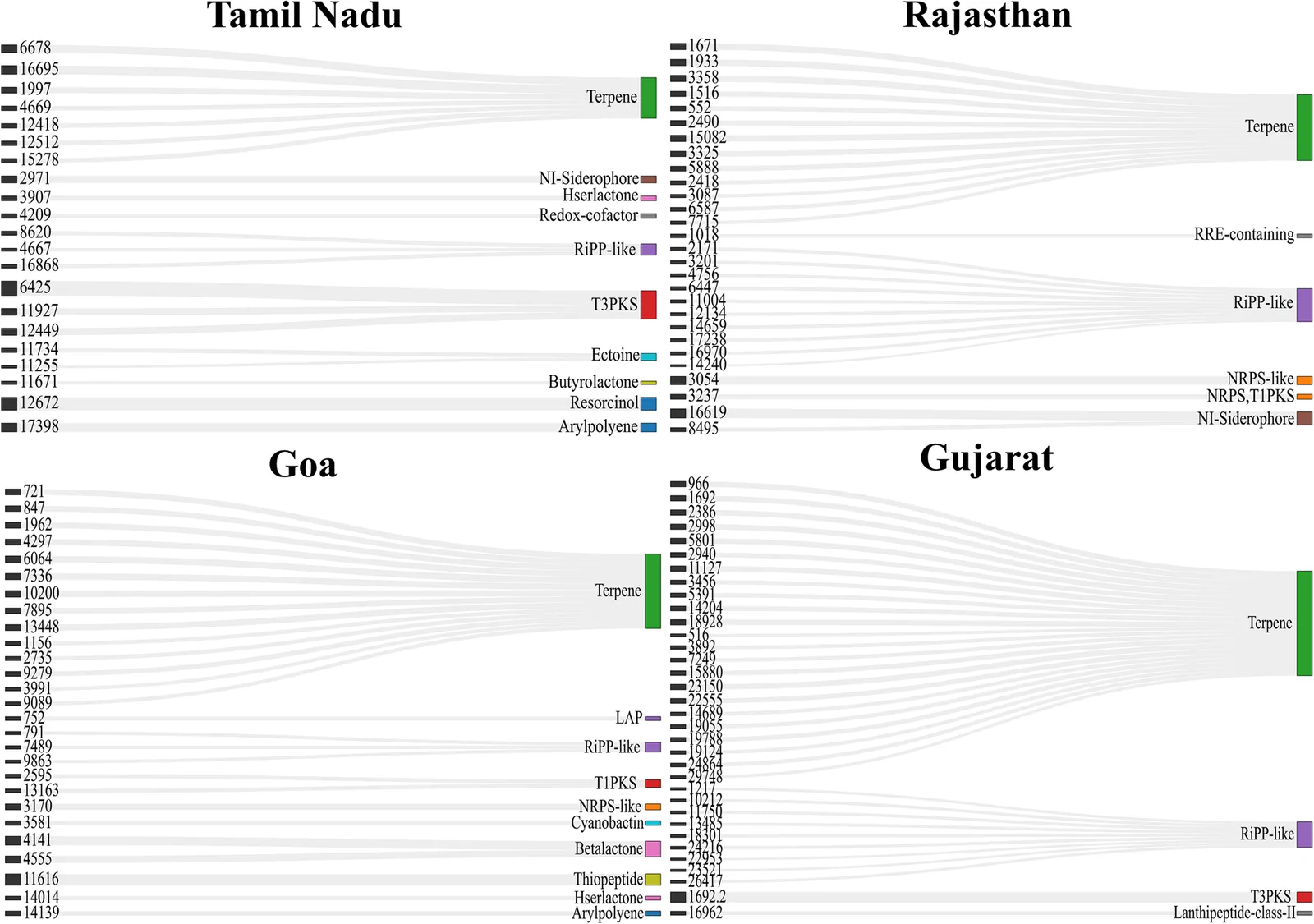
Sankey diagram illustrating the distribution of biosynthetic gene clusters across different biosynthetic types. The left side of the plot shows individual BGCs (labelled by their corresponding genomic regions), while the right side represents the biosynthetic types (e.g., terpenes, NRPS, RiPPs like, etc.). The thickness of the links between clusters and biosynthetic types indicates the magnitude of the biosynthetic potential for that cluster towards a specific type. Node colours distinguish individual clusters and biosynthetic pathway

The distribution of various biosynthetic gene clusters (BGCs) across different biosynthetic pathways, such as terpene, RiPP-like, T3PKS, and others, is represented in Fig. 8. In Rajasthan, a variety of biosynthetic pathways were detected, with a dominance of terpene and RiPP-like clusters, indicating a substantial genetic potential for secondary metabolite production. Terpenes contribute to oxidative stress protection, membrane stabilization, and antimicrobial activity, with applications in pharmaceuticals and cosmetics [77, 78]. RiPP-like peptides are frequently associated with microbial competition and antimicrobial defense, representing promising scaffolds for novel antibiotic discovery. Type III polyketide synthases (T3PKS) support the biosynthesis of pigments and stress-responsive metabolites that enhance UV and stress tolerance [79]. Tamil Nadu also showed a broad array of biosynthetic regions, including terpenes, ectoine, and NI-siderophores, with notable sequences linked to ectoine, butyrolactone, and polyketides, suggesting adaptations related to osmotic and environmental stress. NI-siderophores facilitate iron acquisition in saline, nutrient-poor environments and hold potential for applications in agriculture and bioremediation, while ectoine is widely exploited for enzyme stabilization and biopharmaceutical formulations [80].

Similarly, Goa demonstrated a comparable trend, with a high prevalence of terpene-associated pathways, along with the identification of cyanobactins and beta lactones, which have been previously associated with antimicrobial activity. Gujarat exhibited a particularly strong representation of terpene biosynthesis, along with several RiPP-like clusters, indicating the potential for diverse secondary metabolite production. Across all regions, several gene clusters showed high similarity to known biosynthetic pathways, including carotenoids and polyketides, confirming the presence of well-characterized secondary metabolite-producing pathways. Collectively, these patterns indicate that Indian saltern and Sambhar Salt Lake ecosystems harbor microorganisms with both conserved and potentially novel biosynthetic capabilities. This diversity points to promising opportunities for further exploration of these environments to discover bioactive compounds relevant to biotechnology, pharmaceuticals, and agriculture.

### Integration of culture-dependent and metagenomic findings

Upon comparative evaluation between the culture-dependent isolates (Section Identification and phylogenetic analysis of culturable strains) and the whole-metagenome taxonomic annotations (Section  Microbial taxonomy annotation) expected but significant compositional divergence was observed, this highlights the complementary value of the integrated approach. While the culture-independent metagenomic profiles were heavily dominated by *Halorubrum*, *Halomicroarcula*, *Haloarcula*, and *Halobellus*, our culturomic efforts predominantly recovered *Haloarcula*, *Haloferax*, and members of the Firmicutes (e.g., *Bacillus*, *Halobacillus*). This discrepancy underscores well-documented cultivation biases, wherein highly abundant, slow-growing environmental specialists (like *Halorubrum*) are frequently outcompeted in vitro by opportunistic, fast-growing taxa that thrive under nutrient-rich laboratory conditions. Consequently, while metagenomics accurately captured the true in situ ecological architecture of the hypersaline brines, the targeted culturomics approach successfully enriched a specific sub-population of biotechnologically tractable, multi-enzymatic strains that would have otherwise been overshadowed in the total environmental read counts. We emphasize that the dominant genera recovered through culture (e.g., *Haloarcula*, *Haloferax*, *Bacillus*) differ substantially from those dominating the metagenomic profiles (e.g., *Halorubrum*, *Halomicroarcula*), and the two datasets should therefore be regarded as complementary rather than mutually validating.

Functionally, the enzyme activities detected in plate-based assays (amylase, protease, lipase, esterase, xylanase, cellulase; Supplementary Table S2) were independently corroborated by MMseqs2 annotations of the metagenomic contigs, which recovered the corresponding glycoside hydrolase, peptidase, and lipase gene families across all four sites, with the highest gene counts in the Gujarat and Rajasthan samples, the same samples from which the most active hydrolytic isolates were obtained.

Physicochemically, the distinct environmental parameters summarised in Table 1 aligns with the observed shift in community composition. The extreme condition recorded for Gujarat and Rajasthan matrices coincided with both the archaeal dominance observed in culture and the elevated Halobacteria read fractions recovered by metagenomics. Separately, the specific physicochemical profiles of the Goa sediment and Tamil Nadu liquid brine supported a broader bacterial presence in both datasets. Taken together, the two approaches are complementary rather than parallel i.e. culture isolates provided phenotypic alignment with the predictive functional potential inferred from the metagenomes, while the metagenomic data extended the taxonomic and functional picture beyond what could be recovered on laboratory media.

We acknowledge several limitations in this study. First, our single-season sampling strategy lacked non-saline environmental controls for baseline comparison. Second, because each geographic site was represented by a different sample matrix (e.g., liquid brine, sediment, or crystalline salt), we cannot definitively separate the effects of geography, local chemistry, and matrix type on the microbiome. Finally, without isolate whole-genome sequencing or metagenome-assembled genomes (MAGs), we cannot directly link our cultured isolates to environmental genes at the strain level. Future work will address these gaps through multi-season sampling, standardized matrix comparisons, temperature-gradient enrichment, and genome-resolved metagenomics.

## Conclusion

This study demonstrates that Indian solar salterns serve as natural evolutionary laboratories where salinity-driven selection shapes microbial community assembly, physiology, and biosynthetic potential. By integrating culturomics with whole-metagenome profiling across four climatically and geochemically distinct salterns, we show that these hypersaline habitats harbor a reproducible core of halophilic archaea and bacteria whose distribution is strongly associated with salinity, pH, and conductivity. The significance of the work lies in three directions. First, it establishes an ecological baseline for Indian solar salterns, linking community structure to measurable physicochemical drivers and providing a reference framework against which future studies of hypersaline microbiomes, including those under climate and land-use change, can be compared. Second, the recovery of taxonomically diverse isolates with amylolytic, proteolytic, lipolytic, xylanolytic, and cellulolytic activity, corroborated by the corresponding gene families in the metagenomes, suggests that these environments are potential source of salt-, solvent-, and temperature-tolerant extremozyme candidates of potential value to food, pharmaceutical, textile, leather, and bioremediation industries. Third, the detection of multiple biosynthetic gene clusters, including non-ribosomal peptide synthetase and polyketide synthase loci, points to underexplored secondary-metabolite chemistry that warrants dedicated discovery efforts. Collectively, the findings reinforce the broader purpose of studying extreme environments, not merely to catalog novelty, but to understand how life is organized under physicochemical limits and to translate that understanding into sustainable biotechnology.

## Supplementary Information

Supplementary Material 1.

## Data Availability

The datasets generated and/or analysed during the current study are available in public repositories. The 16S rRNA sequences generated from culturomics are available in GenBank; the individual accession numbers for all isolates are detailed in Supplementary Table S1. The raw whole-metagenome sequencing data generated in this study are available in the NCBI Sequence Read Archive (SRA) database under the BioProject accession number PRJNA1444485.

## Abbreviations

MHM: Moderate Halophilic Media
EHM: Extreme Halophilic Media
HiFi: High-Fidelity
AMR: Antimicrobial Resistance
PCoA: Principal Coordinate Analysis
COG: Cluster of Orthologous Gene
BGC: Biosynthetic Gene Clusters
RiPP: Ribosomally synthesized and post-translationally modified peptides
T3PKS: Type III polyketide synthases
