# Supplementary material for: Eco-technological potential of salinity-driven functional specialization in Indian solar salterns revealed by integrated culturomics and whole-metagenome profiling
Source: BMC Microbiol. 2026 Jun 20;26:818. doi: 10.1186/s12866-026-05253-8 (PMC13587509; doi:10.1186/s12866-026-05253-8)
Supplement: Supplementary file 1 [file 12866_2026_5253_MOESM1_ESM.docx]

**Eco-Technological Potential of Salinity-Driven Functional Specialization in Indian Solar Salterns Revealed by Integrated Culturomics and Whole-Metagenome Profiling**

Shubham Pandey^1^, Bhavna Parmar^1^, Anjali Gupta^1^, Ayushi Singh^1^, Ashwini Chauhan^1^, Kuo-Wei Huang^2^*, Ram Karan^1^*

^1^Department of Microbiology, University of Delhi South Campus, New Delhi

^2^Center for Renewable Energy and Storage Technologies (CREST), KAUST Catalysis Center (KCC), Physical Science and Engineering Division, King Abdullah University of Science and Technology, Thuwal, 23955 Saudi Arabia

*Corresponding author: R. Karan ([ramkaran@south.du.ac.in](mailto:ramkaran@south.du.ac.in)); K. W. Huang ([kuowei.huang@kaust.edu.sa](mailto:kuowei.huang@kaust.edu.sa))

**SUPPLEMENTARY FILE**

**Table of Contents**

| **Item** | **Title / Description** | **Page No.** |
| --- | --- | --- |
| Figure S1 | TapeStation 4150 profiles of library loaded in Agilent High Sensitivity D1000ScreenTape® | 2 |
| Figure S2 | Metagenomic rarefaction curves for the sampled hypersaline environments. | 3 |
| Figure S3 | Heatmap of the top phyla found in different samples (OTU abundance). | 4 |
| Figure S4 | Hierarchical clustering heatmap of halophilic microbial species. | 5 |
| Table S1 | Molecular identification of organisms (16S rRNA) and GenBank accession numbers. | 6-8 |
| Table S2 | Enzymatic characterization of the isolates (Zone of hydrolysis). | 9-10 |
| Table S3 | Sequencing, Assembly, and Gene Prediction Statistics | 11 |
| Table S4 | Culturable phenotypic diversity versus metagenomic relative abundance of microbial genera across the sampled environments | 11 |
| Table S5 | Comparative COG functional profiling and absolute gene counts across four hypersaline microbiomes. | 12 |

**Supplementary Files**

**Supplementary Figure S1:** TapeStation 4150 profiles of library loaded in Agilent High Sensitivity D1000ScreenTape®

**Supplementary Figure S2.** Metagenomic rarefaction curves for the sampled hypersaline environments**.** The curves depict the number of observed taxonomic features (genera) as a function of sequencing depth (number of high-quality reads) for the samples from Tamil Nadu, Rajasthan, Goa, and Gujarat. Analysis was generated using the vegan package in R.

**Supplementary Figure S3.** Heatmap of the top phyla found in different samples. Operational Taxonomic Units were created according to the abundance of genes found in the sample. The OTUs were further matched across various databases to obtain the number of hits, which are shown by numerical values, with the colour gradient from blue (low abundance) to red (high abundance).

**Supplementary Figure S4.** Hierarchical Clustering Heatmap of Halophilic Microbial Species Across Different Environments. The number of hits is shown by numerical values, with the colour gradient from red to blue representing the observed abundance of hits. (The bacterial species are represented by green colour and Archaea by black colour).

**Supplementary Table 1**. Biochemical and Molecular characterization of the identified organisms across various sites with their most identical neighbour along with their accession number*.*

| **Location** | **Kingdom** | **Phylum** | **Genus** | **Strain name** | **Accession no. (Genbank)** | **Gram’s Reaction** | **Cell morphology** | **Pigmentation** |
| --- | --- | --- | --- | --- | --- | --- | --- | --- |
| Tamil Nadu | Archaea | Euryarchaeota | *Haloferax* | XBL22 | PQ895653 | - | Short rod | Red |
|  |  |  | *Halobacillus* | XBL41 | PV034406 | + | Short rod | Cream |
|  |  |  |  | XBL42 | PV034408 | + | Short rod | Creamy |
| Rajasthan | Bacteria | Firmicutes | *Thalassobacillus* | XBL25 | PV034025 | + | Short rod | Cream |
|  |  |  | *Halobacillus.* | XBL37 | PV034371 | + | Short rod | Creamy |
|  |  |  | *Gracilibacillus* | XBL26 | PV034039 | + | Short rod | Pale yellow |
| Goa | Bacteria | Firmicutes | *Salibacterium* | XBL1 | PQ892033 | + | Short rod | White/creamy |
|  |  |  |  | XBL3 | PQ892058 | + | Short rod | White/creamy |
|  |  |  |  | XBL4 | PQ892063 | + | Short rod | White/creamy |
|  |  |  | *Bacillus* | XBL6 | PQ895561 | + | Short rod | Off White |
|  |  |  | *Gracilibacillus* | XBL39 | PV034384 | + | Short rod | Pale yellow |
|  |  |  | *Virgibacillus* | XBL38 | PV034376.1 | + | Short rod | Pale yellow |
|  | Archaea | Euryarchaeota | *Haloarcula* | XBL2 | PQ892057 | - | Irregular rods | Orange |
|  |  |  | *Haloferax* | XBL5 | PQ895565 | - | Short rod | Red |
|  |  |  |  | XBL40 | PV034387 | - | Short rod | Red |
| Gujarat | Bacteria | Firmicutes | *Marinococcus* | XBL30 | PV034303 | + | Cocci | Creamy |
|  |  |  |  | XBL27 | PV034260 | + | Cocci | Creamy |
|  |  |  |  | XBL33 | PV034335 | + | Cocci | Creamy |
|  |  |  | *Salicola* | XBL24 | PV012702 | - | Cocci | Light red |
|  | Archaea | Euryarchaeota | *Haloarcula* | XBL7 | PQ895599 | - | Irregular rods | Orange |
|  |  |  |  | XBL8 | PQ895600 | - | Irregular rods | Orange |
|  |  |  |  | XBL12 | PQ892062 | - | Irregular rods | Orange |
|  |  |  |  | XBL13 | PQ895624 | - | Irregular rods | Orange |
|  |  |  |  | XBL14 | PX850706 | - | Irregular rods | Reddish Orange |
|  |  |  |  | XBL15 | PQ895626 | - | Irregular rods | Orange |
|  |  |  |  | XBL16 | PQ895650 | - | Irregular rods | Orange |
|  |  |  |  | XBL17 | PQ895641 | - | Irregular rods | Orange |
|  |  |  |  | XBL18 | PQ895643 | - | Irregular rods | Orange |
|  |  |  |  | XBL20 | PQ895657 | - | Irregular rods | Orange |
|  |  |  |  | XBL21 | PQ895652 | - | Irregular rods | Orange |
|  |  |  |  | XBL29 | PV034280 | - | Irregular rods | Orange |
|  |  |  |  | XBL31 | PV034308 | - | Irregular rods | Orange |
|  |  |  |  | XBL32 | PV034309 | - | Irregular rods | Orange |
|  |  |  |  | XBL34 | PV034338 | - | Irregular rods | Orange |
|  |  |  |  | XBL35 | PV034346 | - | Irregular rods | Orange |
|  |  |  |  | XBL36 | PV034358 | - | Irregular rods | Orange |
|  |  |  | *Haloferax* | XBL9 | PX854917 | - | Short rod | Red |
|  |  |  |  | XBL10 | PQ895601 | - | Short rod | Red |
|  |  |  |  | XBL11 | PQ895605 | - | Short rod | Red |
|  |  |  |  | XBL19 | PX854914 | - | Short rod | Red |
|  |  |  |  | XBL23 | PQ932525 | - | Short rod | Red |
|  |  |  |  | XBL28 | PV034279 | - | Short rod | Red |

**Supplementary Table 2**. Enzymatic characterization of the isolates. Semi-quantitative enzyme activity of isolates measured as halo zone diameter (mm) after subtracting colony diameter. Values represent mean ± standard deviation (n = 3). “–” indicates no detectable activity. Due to the plate-based assay format, measurements are semi-quantitative and reflect relative enzyme production.

| **Strain name** | **Accession no. (Genbank)** | **Zone of hydrolysis (in mm)** | | | | | |
| --- | --- | --- | --- | --- | --- | --- | --- |
|  |  | **Amylase** | **Esterase** | **Protease** | **Xylanase** | **Lipase** | **Cellulase** |
| XBL1 | PQ892033 | 3.0 ± 0.62 | 4.0 ± 0.48 | – | – | 4.0 ± 0.61 | – |
| XBL2 | PQ892057 | – | 3.5 ± 0.54 | – | – | – | – |
| XBL3 | PQ892058 | – | 3.0 ± 0.43 | – | – | – | – |
| XBL4 | PQ892063 | – | 3.0 ± 0.28 | 4.0 ± 0.61 | – | 4.0 ± 0.43 | – |
| XBL5 | PQ895565 | – | 3.6 ± 0.31 | – | – | – | – |
| XBL6 | PQ895561 | 4.0 ± 0.82 | 4.0 ± 0.46 | – | – | 3.0 ± 0.47 | – |
| XBL7 | PQ895599 | 4.0 ± 0.54 | 6.0 ± 0.86 | – | – | – | – |
| XBL8 | PQ895600 | 3.0 ± 0.45 | 4.0 ± 0.67 | – | – | – | – |
| XBL9 | PX854917 | 8.2 ± 0.97 | 3.1 ± 0.23 | – | 1.8 ± 0.19 | – | – |
| XBL10 | PQ895601 | – | – | – | 2.7 ± 0.39 | – | – |
| XBL11 | PQ895605 | – | – | – | – | 1.4 ± 0.18 | – |
| XBL12 | PQ892062 | 7.0 ± 0.79 | 9.0 ± 1.25 | 4.0 ± 0.47 | – | – | – |
| XBL13 | PQ895624 | – | 3.0 ± 0.49 | – | – | – | – |
| XBL14 | PX850706 | 1.0 ± 0.14 | 4.0 ± 0.49 | – | – | – | – |
| XBL15 | PQ895626 | 5.0 ± 0.69 | 9.0 ± 1.08 | – | – | – | – |
| XBL16 | PQ895650 | 3.0 ± 0.43 | – | 2.0 ± 0.36 | – | – | – |
| XBL17 | PQ895641 | – | – | 2.3 ± 0.32 | 2.6 ± 0.33 | 1.8 ± 0.24 | – |
| XBL18 | PQ895643 | 1.4 ± 0.19 | – | – | – | – | – |
| XBL19 | PX854914 | 2.2 ± 0.24 | 4.2 ± 0.68 | – | 3.0 ± 0.41 | – | – |
| XBL20 | PQ895657 | 1.8 ± 0.22 | – | – | – | – | – |
| XBL21 | PQ895652 | – | 8.2 ± 0.97 | – | – | – | – |
| XBL22 | PQ895653 | 1.5 ± 0.20 | – | – | – | 1.4 ± 0.17 | – |
| XBL23 | PQ932525 | – | 5.0 ± 0.71 | – | – | – | – |
| XBL24. | PV012702 | 9.0 ± 1.15 | 8.0 ± 1.02 | 5.0 ± 0.81 | – | – | – |
| XBL25 | PV034025 | – | 4.0 ± 0.79 | – | – | – | 7.0 ± 0.86 |
| XBL26 | PV034039 | – | – | 8.0 ± 1.11 | – | 5.0 ± 0.47 | 5.0 ± 0.67 |
| XBL27 | PV034260 | – | – | 7.0 ± 0.82 | – | – | – |
| XBL28 | PV034279 | – | 7.0 ± 0.67 | 6.0 ± 0.78 | – | – | – |
| XBL29 | PV034280 | 7.0 ± 0.47 | – | – | – | 5.0 ± 0.72 | 3.0 ± 0.44 |
| XBL30 | PV034303 | – | 7.0 ± 0.40 | 6.0 ± 0.73 | – | – | 4.0 ± 0.46 |
| XBL31 | PV034308 | 6.0 ± 0.81 | – | – | – | – | 4.0 ± 0.52 |
| XBL32 | PV034309 | – | 4.0 ± 0.63 | 6.0 ± 0.86 | – | 7.0 ± 0.97 | – |
| XBL33 | PV034335 | – | – | 6.0 ± 0.77 | – | – | 3.0 ± 0.41 |
| XBL34 | PV034338 | 6.0 ± 0.84 | 7.0 ± 0.92 | – | – | – | – |
| XBL35 | PV034346 | – | 6.0 ± 0.81 | 7.5 ± 0.53 | – | – | – |
| XBL36 | PV034358 | 9.0 ± 1.36 | 9.0 ± 1.25 | – | – | – | – |
| XBL37 | PV034371 | – | – | 8.0 ±0.98 | – | 5.0 ± 0.42 | 5.0 ± 0.68 |
| XBL38 | PV034376.1 | – | – | 7.5 ± 0.97 | – | – | – |
| XBL39 | PV034384.1 | – | – | 9.0 ± 1.09 | – | – | – |
| XBL40 | PV034387 | – | 3.0 ± 0.43 | – | – | – | – |
| XBL41 | PV034406 | 6.0 ± 0.57 | 3.0 ± 0.12 | – | – | – | – |
| XBL42 | PV034408 | 7.0 ± 0.81 | 5.0 ± 0.52 | – | – | – | – |

**Supplementary Table S3.** Sequencing, Assembly, and Gene Prediction Statistics

| **Sample** | **PE Reads** | **Total reads (R1+R2)** | **Total bases (R1+R2)** | **Total data (Gb)** | **Scaffolds** | **Total scaffold length (bp)** | **Average scaffold length (bp)** | **Max scaffold size (bp)** | **No. of genes** | **Total genes size (bp)** | **Average gene size (bp)** | **Max gene size (bp)** |
| --- | --- | --- | --- | --- | --- | --- | --- | --- | --- | --- | --- | --- |
| **Tamil Nadu** | 15,210,271 | 30,420,542 | 4,836,866,178 | 4.84 | 323,846 | 281,807,200 | 870.2 | 65,322 | 503,796 | 251,450,169 | 499.1 | 14,241 |
| **Rajasthan** | 19,569,793 | 39,139,586 | 6,223,194,174 | 6.22 | 311,970 | 283,476,374 | 908.7 | 335,560 | 517,927 | 248,314,932 | 479.4 | 22,812 |
| **Goa** | 16,720,776 | 33,441,552 | 5,317,206,768 | 5.32 | 245,509 | 233,307,380 | 950.3 | 416,255 | 429,420 | 206,857,023 | 481.7 | 12,081 |
| **Gujarat** | 27,285,025 | 54,570,050 | 8,676,637,950 | 8.68 | 357,604 | 380,909,309 | 1,065.2 | 383,639 | 669,991 | 331,249,545 | 494.4 | 13,272 |

**Supplementary Table S4.** Culturable phenotypic diversity versus metagenomic relative abundance of microbial genera across the sampled environments.

| **Location** | **Recovered Culturable Genera** | **Top Metagenomic Genera (Relative Abundance %)** |
| --- | --- | --- |
| **Tamil Nadu** | *Halobacillus* (2), *Haloferax* (1) | *Marinobacter* (5.67%), *Rhodohalobacter* (5.19%), *Halomonas* (4.13%) |
| **Rajasthan** | *Thalassobacillus* (1), *Halobacillus* (1), *Gracillibacillus* (1) | *Natronococcus* (3.77%), *Natronorubrum* (2.40%), *Natrinema* (1.70%) |
| **Goa** | *Salibacterium* (3), *Haloferax* (2), *Haloarcula* (1), *Bacillus* (1), *Gracillibacillus* (1), *Virgibacillus*(1) | *Halomicroarcula* (10.50%), *Bradyrhizobium* (4.26%), *Haloplanus* (4.17%) |
| **Gujarat** | *Haloarcula* (17), *Haloferax* (6), *Marinococcus* (3), *Salicola* (1) | *Halorubrum* (12.66%), *Halobellus* (4.91%), *Halonotius* (4.41%), *Haloarcula* (3.90%)* |

* Only Gujarat sample yielded a culturable genera abundant in the metagenomic data as well.

(It is important to note that the culture-dependent results represent recoverable phenotypic diversity rather than quantitative abundance. During isolation, representative morphotypes were selected to maximize the discovery of distinct genera, thereby screening out redundant colonies. Consequently, while metagenomics provides a true measure of relative abundance in the environmental sample, the culture-dependent data highlights the specific genera that were viable and maintained in laboratory cultivation under the applied conditions.)

**Supplementary Table S5**: Comparative COG functional profiling and absolute gene counts across four hypersaline microbiomes.

| **Description** | **Tamil Nadu** | **Rajasthan** | **Goa** | **Gujarat** |
| --- | --- | --- | --- | --- |
| RNA processing and modification | 76 | 77 | 48 | 55 |
| Chromatin structure and dynamics | 261 | 196 | 147 | 278 |
| Energy production and conversion | 21625 | 20689 | 16444 | 17713 |
| Cell cycle control, cell division, chromosome partitioning | 3018 | 2466 | 2058 | 3164 |
| Amino acid transport and metabolism | 26891 | 26564 | 21426 | 26506 |
| Nucleotide transport and metabolism | 7461 | 6824 | 5561 | 8193 |
| Carbohydrate transport and metabolism | 13059 | 11217 | 9823 | 9398 |
| Coenzyme transport and metabolism | 9749 | 9718 | 7864 | 10219 |
| Lipid transport and metabolism | 11792 | 10403 | 8556 | 8690 |
| Translation, ribosomal structure and biogenesis | 17899 | 14927 | 12680 | 19528 |
| Transcription | 12155 | 12126 | 12200 | 16463 |
| Replication, recombination and repair | 17846 | 21059 | 17051 | 32554 |
| Cell wall/membrane/envelope biogenesis | 16280 | 11384 | 9599 | 12142 |
| Cell motility | 4951 | 3650 | 3583 | 4570 |
| Posttranslational modification, protein turnover, chaperones | 12724 | 10413 | 9098 | 12105 |
| Inorganic ion transport and metabolism | 15034 | 12199 | 12538 | 13478 |
| Secondary metabolites biosynthesis, transport and catabolism | 5190 | 4340 | 3714 | 3842 |
| General function prediction only | 32025 | 32188 | 28315 | 37244 |
| Function unknown | 17800 | 19061 | 18547 | 23372 |
| Signal transduction mechanisms | 13370 | 11460 | 11226 | 12696 |
| Intracellular trafficking, secretion, and vesicular transport | 3331 | 2514 | 2510 | 2941 |
| Defense mechanisms | 5821 | 4558 | 3863 | 6369 |
| Cytoskeleton | 147 | 10 | 5 | 26 |
